# Iontophoresis Drug Delivery to the Macula of Lutein for Age Related Macular Degeneration

**DOI:** 10.3390/diseases14090310

**Published:** 2026-08-26

**Authors:** Maria Letizia Salvetat, Francesco Pellegrini, Marco Zeppieri, Matteo Capobianco, Alessandro Avitabile, Ludovica Cannizaro, Giuseppe Gagliano, Francesco Cappellani, Caterina Gagliano

**Affiliations:** 1 Department of Ophthalmology, Azienda Sanitaria Friuli Occidentale, 33170 Pordenone, Italy; mlsalvetat@hotmail.it (M.L.S.);; 2 Department of Ophthalmology, University Hospital of Udine, 33100 Udine, Italy; 3 Department of Medicine, Surgery and Health Sciences, University of Trieste, 34129 Trieste, Italy; 4 Eye Clinic, Catania University, Policlinico G. Rodolico, 95121 Catania, Italy; 5 Biomedicine, Neuroscience and Advance Diagnostic (BIND) Department, University of Palermo, 90128 Palermo, Italy; 6 Department of Medicine and Surgery, University of Enna “Kore”, Piazza dell ’Università, 94100 Enna, Italy; 7 Mediterranean Foundation “G.B. Morgagni”, 95125 Catania, Italy

**Keywords:** age-related macular degeneration, lutein, macula, iontophoresis, retina

## Abstract

Background: Age-related macular degeneration (AMD) represents a leading cause of irreversible vision loss worldwide, driven by intricate interactions among oxidative stress, low-degree chronic inflammation, and macular pigment depletion in aging retinas. Lutein, a component of the macular pigment, has attracted significant attention in AMD management for its antioxidant and blue-light filtering properties. Its oral supplementation, however, although showing potential in slowing AMD progression, requires daily intake and results in variable macular absorption. Trans-scleral iontophoresis (TSI) is a non-invasive technique using low-intensity electric currents to deliver charged molecules into biological tissues and represents an innovative method to deliver lutein into the retina. Methods: The present narrative review synthesizes and discusses the results of lutein supplementation in AMD patients; the ocular iontophoresis basal principles, efficacy, safety and limitations in treating different ophthalmic pathologies; and the evolving clinical evidence supporting lutein TSI in the AMD management. Results: *Ex-vivo* studies have shown that lutein TSI may provide efficient, localized, rapid, and sustained retinal supplementation of macular pigment, overcoming oral intake limitations. Recent preliminary clinical studies have demonstrated that this technique is well-tolerated and effective in enhancing macular pigment optical density and improving some visual functions in AMD patients. Conclusions: *Ex-vivo* and pilot clinical studies highlight the capacity of TSI to deliver lutein inside the retina, overcoming physiological barriers that may limit the efficacy of lutein oral administration. The demonstration of the efficacy of the procedure in preserving/improving visual functions and delaying disease progression in AMD patients requires further long-term randomized controlled clinical studies.

## 1. Introduction

Age-related macular degeneration (AMD) represents the most common aging eye disease [1,2] and one of the leading causes of irreversible visual loss in people older than 50 years worldwide [3,4]. The most endorsed pathophysiological mechanisms explaining its onset and progression have been linked to oxidative stress, low-degree chronic inflammation, and macular pigment depletion in aging retinae [5,6]. The macular pigment is composed by carotenoids, i.e., yellow to red coloring substances synthetized by plants, algae, bacteria, yeast and molds, whose correct dietary intake is essential for the correct functioning of several human body organs, including the eye [7]. The specific role of macular carotenoids seems to be the protection of retinal integrity from the light-induced oxidative damage [8,9] and their reduced dietary intake or age-related macular depletion have been associated to AMD prevalence [10] or progression [11].

Lutein represents the most abundant carotenoid and the most efficient blue light filter present in the human eye [12,13] and its dietary supplementation has shown potential in slowing down AMD progression to late stages, as confirmed by large-scale randomized clinical trials [14]. Nevertheless, its effective delivery into the retina after oral administration remains challenging because of the need of daily assumption, which is dependent on patient’s compliance [15,16], and the variability of its effective bioavailability, which is related to many dietary, metabolic and genetic factors [17]. Iontophoresis is a technique that uses low-intensity electric currents to deliver ionized or charged molecules to a target [18]. Widely used in medical practice since 1993 [19] in order to transport specific drugs into biological tissues [20], over the last two decades, iontophoresis has been employed in ophthalmology as a non-invasive drug delivery system [21,22,23,24,25].

Experimental studies on donor human eye globes have shown that trans-scleral iontophoresis of lutein is able to provide a rapid, efficient, localized, and sustained increase in the macular pigment, thus overcoming the oral supplementation limitations [26,27]. Recently, pilot clinical studies have investigated the efficacy and safety of lutein supplementation via trans-scleral iontophoresis in AMD patients, with promising preliminary results [28,29,30,31]. This narrative review synthesizes the results of lutein supplementation in AMD patients, focusing on emerging research on trans-scleral iontophoretic lutein retinal delivery. Basal principles of iontophoresis, its efficacy, safety, and limitations as a drug delivery system for treating different ophthalmic pathologies are reported. This is the first review summarizing, analyzing, and discussing the evolving clinical evidence supporting trans-scleral iontophoresis for lutein retinal delivery in AMD management.

## 2. Literature Search Strategy and Evidence Synthesis

A structured literature search was conducted in PubMed/MEDLINE up to 5 August 2026. The database search was supplemented by screening the reference lists of relevant original articles, systematic reviews, meta-analyses, and narrative reviews to identify additional publications. The primary search terms were “age-related macular degeneration”, “AMD”, “dry AMD”, “intermediate AMD”, “lutein”, “zeaxanthin”, “carotenoids”, “macular carotenoids”, “macular xanthophylls”, “macular pigment”, “macular pigment optical density”, antioxidant supplementation”, “iontophoresis”, “ocular iontophoresis”, “ophthalmic iontophoresis”, “transscleral iontophoresis”, “trans-scleral iontophoresis”, “posterior segment drug delivery”, “ocular drug delivery”, and “retinal drug delivery”.

Additional targeted searches were conducted using the terms “iontophoresis” or “iontophoretic”, combined with the active compounds, drugs, or diseases being investigated.

Only English-language publications were considered, with no publication date restriction. The absence of a date restriction allowed the inclusion of papers describing the fundamental principles and early ophthalmic applications of iontophoresis, in addition to more recent experimental and clinical evidence concerning trans-scleral lutein delivery. Eligible publications included in vitro, ex vivo, and animal studies; cross-sectional, case–control, and cohort studies; prospective and retrospective clinical studies; pilot interventional studies; controlled and randomized clinical trials; systematic reviews; meta-analyses; and relevant narrative reviews. Studies were eligible for inclusion if they addressed at least one of the following topics: biological actions and retinal distribution of lutein; macular pigment and AMD; oral lutein or carotenoid supplementation; drug delivery to the posterior segment of the eye; principles, pharmacokinetics, efficacy, or safety of ocular iontophoresis; experimental delivery of drugs or other macromolecules through ocular tissues; or transscleral iontophoretic delivery of lutein in experimental or clinical settings.

Exclusion criteria included studies not directly related to lutein, AMD, macular pigment, ocular iontophoresis, or posterior-segment drug delivery; studies lacking sufficient methodological or outcome data, including conference abstracts and isolated case reports. Non-ophthalmic studies were considered only when they provided essential information regarding the general mechanisms or technological principles of an iontophoresis drug delivery system.

For the purposes of evidence classification, experimental studies were defined as in vitro, ex vivo, in vivo, or animal studies evaluating drug transport, tissue penetration, ocular distribution, uptake, pharmacokinetics, or biological, therapeutic, or electrophysical effects. Observational studies were defined as cross-sectional, case-control, or longitudinal cohort studies assessing associations among lutein exposure, circulating or retinal carotenoid levels, macular pigment, visual function, and AMD-related outcomes. Clinical studies were defined as human interventional investigations, including pilot studies, uncontrolled prospective studies, comparative studies, controlled clinical trials, and randomized clinical trials evaluating oral supplementation or iontophoresis-based treatments. Systematic reviews and meta-analyses were classified as secondary evidence, whereas narrative reviews were used primarily to provide context regarding biological mechanisms, technical principles, and historical developments.

Owing to substantial heterogeneity in study populations, AMD stages, lutein formulations and doses, iontophoresis protocols, comparator groups, follow-up periods, and outcome measures, the evidence was synthesized narratively. Greater interpretative emphasis was placed on directly relevant human studies, controlled clinical investigations, systematic reviews, and replicated findings. Experimental studies were used to support biological plausibility and the mechanisms of ocular drug delivery. No formal risk-of-bias assessment was performed. Accordingly, this work was designed as an evidence-based narrative review rather than a systematic review.

## 3. Age-Related Macular Degeneration: A Public Health Burden

Age-related macular degeneration is a chronic, degenerative, and progressive eye disease of multifactorial origin that induces gradual irreversible damage to the macula, resulting in central vision impairment [1,2]. AMD represents the most common aging eye disease and is the most important cause of irreversible vision loss in people older than 50 years in Western countries and one of the leading causes of blindness worldwide [3,4]. Due to rapid population aging and increasing life expectancy in the industrialized world, its prevalence is estimated to increase exponentially in the near future [4,32].

Although not yet completely elucidated, AMD pathophysiology is thought to be complex and multifactorial [5,6], and several risk factors have been associated with AMD onset and progression [33,34]. The most important modifiable risk factors include low dietary intake of vegetables, fruits, and fish and high dietary intake rich in sugar, fat, alcohol, and oils; smoking; systemic hypertension and cardiovascular diseases; hypercholesterolemia and other metabolic co-morbidities; overweight; reduced physical activity; sun and blue light exposure; and previous cataract surgery. Non-modifiable risk factors include: Caucasian race; female sex; positive family history for AMD; genetic variants; dark iris pigmentation; and hyperopic refraction [33,34].

The most endorsed AMD etiopathologic mechanisms involve an intricate relationship among light-induced oxidative damage of the complex photoreceptor/retinal pigment epithelium (RPE)/Bruch’s membrane/choriocapillaris, the so called “blue light hazard” [35], macular pigment depletion [12,36], low-degree chronic inflammation activation [37] in aging [38], and genetically [39] predisposed retinae, where RPE may represent the weak link in the chain [40] (Table A1).

According to disease clinical signs and symptoms, AMD is classified into early, intermediate, and late or advanced stages [1,2,3,4]. Late AMD can be divided into two different types: the dry, non-exudative, or atrophic form (85–90% of cases), characterized by a gradual evolution toward a macular geographic atrophy (GA), and the wet, exudative, or neovascular form (10–15% of cases), which involves the growth of abnormal blood vessels, a process called macular neovascularization (MNV) [3]. Irrespective of the disease variant, patients in the late stage complain of metamorphopsia, central scotomas, and severe visual acuity reduction. Although less prevalent, wet AMD is the cause of 80% of cases of severe vision loss in AMD patients [1,2,3,4].

By compromising central vision, AMD may interfere with several common activities such as driving, reading, face recognition, and work capabilities, thus significantly reducing the quality of life, personal independence, and economic productivity of the affected subjects [4,32]. Because of the enormous productivity loss and health care cost it causes, AMD represents a serious public health challenge [4,32].

AMD is still considered an incurable eye disease, and all currently available treatment strategies are limited to slowing down its progression [5,41]. Intravitreal injections of anti-vascular endothelial growth factor (VEGF) drugs have been successfully used in the last two decades to stop MNV, and are regarded as the gold standard for the management of wet AMD [42]. Conversely, no therapies for prevention or treatment of the early, intermediate, and GA AMD stages have been approved worldwide [43,44]. The main therapeutic approaches to dry AMD are focused on reduction of modifiable risk factors, such as diet modification [45], smoking cessation [46], and physical activity/exercise [47], and on nutritional supplementation with different substances having anti-oxidative, anti-inflammatory, anti-angiogenic, anti-apoptotic, anti-proliferative, or neuroprotective properties [36,48,49,50,51,52]. Intravitreal injections of drugs targeting the complement system cascade, pegcetacoplan and avacincaptad, have been recently approved by the United States (US) Food and Drug Administration (FDA) for the treatment of GA secondary to AMD, but are not yet available in other countries [53].

## 4. Lutein and Its Strategic Role in the Visual Function

### 4.1. The Macular Carotenoids

Lutein is a natural pigment belonging to the chemical class of xanthophylls or oxygenated carotenoids [7,8]. Carotenoids are hydrophobic, lipophilic yellow-to-red coloring pigments synthesized by plants, algae, bacteria, yeast, and molds. Since carotenoids exert several important physiological functions, such as skin and eye photo-protection, antioxidant activity, regulation of the immune system, cell signaling and gene expression, and modulation of several cognitive functions [7,8,36], their correct dietary intake is fundamental for the functioning of specific organs of the body, including the eye.

Although found in many body tissues, carotenoids are greatly concentrated in the retina [7,8]. The macular pigment of the macula lutea or yellow spot is formed by three specific xanthophylls: lutein (35–40% of the macular pigment) and its stereoisomers zeaxanthin (15–20%), which must be introduced with diet [8], and meso-zeaxanthin (10–15%), which is synthesized from lutein into the RPE cells by a specific enzyme (RPE65 isomerase enzyme) [54].

The most common dietary sources of lutein and zeaxanthin are egg yolk, green leafy vegetables, and yellow and orange fruits [7,8]. Lutein and zeaxanthin are fat-soluble molecules that follow the same absorption pathway as dietary lipids. Their final bioavailability to human body tissues has high inter-individual variability due to several dietary, metabolic, and genetic factors [8,17,55,56,57].

### 4.2. Macular Carotenoid Properties (Table A2)

Lutein and zeaxanthin have been demonstrated to exert several important physiological functions, including blue light filtration and anti-oxidative, anti-inflammatory, anti-angiogenic, anti-apoptotic, neuro-modulatory, and anti-aging effects [8,9,12,13,36,55,58,59,60].

However, the physiological accumulation of carotenoids in the retina, with the greatest concentration in the macula lutea, is thought to play a key role in optimizing central visual function and preserving retinal health and function [8,9,12,13,36,55].

The specific task of macular carotenoids seems to be the protection of the complex photoreceptors/RPE/choriocapillaris from blue light-induced oxidative damage via two different pathways: by absorbing the dangerous high-energy blue light, thus preventing reactive oxygen species (ROS) generation; and acting as a scavenger of already formed ROS or other free radicals [8,9,12,13,36,55].

Moreover, likely due to their blue light filtration effect and cerebral visual inputs modulation [9,12], macular carotenoids seem to have a fundamental role in optimizing central visual function, increasing best-corrected visual acuity (BCVA) and contrast sensitivity, and reducing glare, light-scattering, chromatic aberrations, and photo-stress recovery time [8,9,13,36,55].

Lutein, with a maximum absorption peak at 460 nm, seems to be the most efficient blue light filter, whereas zeaxanthin seems to provide a higher antioxidant effect [9,12].

### 4.3. Macular Pigment Optical Density (MPOD)

Macular xanthophyll levels in vivo are usually expressed as macular pigment optical density (MPOD), i.e., the measure of the macular carotenoids’ light absorption capacity [61]. MPOD levels in vivo can be assessed using psychophysical techniques that estimate MPOD by exploring visual perception in response to specific stimulus conditions [62], indirect systemic measurement techniques [61], and objective methods [61,63,64] that provide macular pigment concentration measurements (Table A3).

Giving a measurement of systemic carotenoids, Veggie Meter and High-Performance Liquid Chromatography provide an indirect MPOD measure and are classified as indirect systemic techniques.

Fundus auto-fluorescence provides an MPOD estimation based on the ability of the macular pigment to attenuate the fundus background autofluorescence [61]. However, the eye’s blue light filtering properties are not exclusively related to macular pigment, which reduces the method’s accuracy [61].

Resonance Raman Spectroscopy [64] and Macular Pigment Reflectometry [63] are able to give separate, objective, and accurate measurements of lutein and zeaxanthin concentrations, together with a bi-dimensional map of their distribution in the human retina in vivo, but are very expensive and not available for clinical use [61].

Oral supplementation with macular xanthophylls (lutein ± zeaxanthin) in healthy subjects has been demonstrated to increase MPOD levels in a dose-dependent manner [65,66,67].

The relationship between MPOD levels and visual performance in healthy subjects is still under investigation. Although higher MPOD levels have been shown to be associated with higher contrast sensitivity [68,69,70,71,72] and lower glare disability and photo-stress recovery time [70,73], the relationship between MPOD and BCVA is still controversial.

Previous authors have found a positive correlation between MPOD levels and BCVA [68], and between increased MPOD values due to oral supplementation of macular carotenoids and improved BCVA [66]. Conversely, other studies did not find a direct correlation between MPOD and BCVA [70,71,74].

Moreover, increased MPOD levels due to carotenoid oral supplementation have been found to primarily influence mesopic and scotopic BCVA rather than high-contrast photopic BCVA [71] and to provide BCVA improvements in patients affected by eye diseases but not in healthy subjects with normal MPOD levels at baseline [74].

Finally, MPOD levels have been shown to decrease with age [75], thus likely contributing to the age-related decrease in visual performance.

## 5. The Role of Lutein in AMD Management

Understanding the fundamental role played by photo-oxidative stress and age-related macular pigment depletion in the AMD pathophysiology [5,6] suggests that higher dietary intake or supplementation with macular carotenoids or other specific nutrients may have therapeutic potential.

Moreover, the numerous observational and intervention studies investigating the influence of dietary intake or supplementation of lutein or other substances on AMD onset or progression have provided highly heterogeneous outcomes [14,15,16,36,48,49,50,51,52,55,57,76,77]. Factors advocated to explain study results inconsistencies include different inclusion/exclusion criteria; use of objective or self-reported data; methods used to assess lutein serum concentration, MPOD levels, retinal morphology, visual performance, or AMD stage and progression; type, dose, and duration of oral supplementation; patient’s adherence to oral supplementation; etc.

### 5.1. The Clinical Epidemiological Observational Studies: The Effect of Lutein Dietary Intake (Table A4)

Several observational studies have investigated the impact of different dietary habits on AMD epidemiology [45,78,79,80,81]. However, the relationship between lutein dietary intake and AMD risk is still controversial. Although several studies have demonstrated a significant inverse relationship between higher macular xanthophylls dietary intake and risk of AMD onset [10,82,83,84,85,86,87] or progression [11,86], even in highly genetically predisposed subjects [11,84], other authors did not find any significant association [88,89,90].

Many previous studies have found a significant inverse relationship between lutein serum levels and risk of AMD onset [91,92,93,94] or progression to late stages [95,96], whereas other authors did not find significant differences in lutein blood levels between AMD patients and healthy subjects [88,95,97].

Comparative studies have demonstrated significantly higher MPOD levels in healthy subjects as compared to AMD patients [98,99,100,101] and to first-degree relatives of AMD patients [102], suggesting that low MPOD values may be considered a biomarker of subjects with high-risk of AMD development [61,102,103]. Conversely, other authors did not find significant differences in MPOD values between AMD and healthy patients [104,105,106], and a recent study has shown significantly higher MPOD levels in AMD patients than in healthy subjects [107].

### 5.2. The Clinical Intervention Studies: The Effect of Lutein Oral Supplementation

The average daily lutein dietary consumption of adults in Western countries ranges between 0.5 and 4 mg, with a median of 1–2 mg [7]. Even though the optimal dosage is still under discussion, a protective effect against AMD has been associated with oral lutein supplementation ranging between 10 mg and 20 mg per day over periods longer than 3–6 months, whereas dosages lower than 10 mg/day for treatment durations shorter than 6 months have not provided significant benefits [15,16]. Furthermore, several lutein supplementation studies have demonstrated an overflow phenomenon, i.e., the initial increase in the lutein plasma concentration is followed by a decrease despite continuous supplementation, which is thought to be related to the downregulation of lutein receptor scavengers in the gastrointestinal tract or to an upregulation of lutein-degrading enzyme β-carotene dioxygenase [108].

#### 5.2.1. The Influence of Lutein Oral Supplementation on Lutein Plasma and Macular Levels (MPOD) and Visual Performance in AMD Patients

Several RCTs have demonstrated that oral supplementation of lutein ± zeaxanthin and other antioxidants in AMD patients may induce a significant increase in lutein serum concentration [109,110,111,112,113]. On the other hand, the direct conversion of lutein oral supplementation into an MPOD level increase is still controversial, with some authors supporting [109,110,114,115,116,117,118,119] and others denying this assumption [111,112,120,121] (Table A5).

Large meta-analyses have shown that a 2-month daily oral intake of 10 mg of lutein, which represents the most common dose found in commercial products, may increase lutein plasma concentration by 3–5 times and MPOD by approximately 5% in both healthy and AMD patients, and that its discontinuation may lead to a pre-treatment plasma level within 1–4 months, whereas MPOD levels remain elevated for longer periods [15,16,61,103].

Whether or not a lutein plasma or macular concentration increase due to lutein oral supplementation can directly translate into visual performance improvement is still under investigation. Many studies have demonstrated that the MPOD increases due to oral supplementation with lutein ± other anti-oxidants are associated with significant BCVA improvement/stabilization [109,114,117] and enhanced contrast sensitivity [110,114,116,118,121], glare recovery [114], microperimetric sensitivity [115], or multifocal electroretinogram [ERG] parameters [116,122], especially in early-stage AMD patients [109,110,116,121]. On the other hand, other authors did not find any significant correlation between MPOD increase and visual performance modification [119,123], suggesting that MPOD enrichment may be necessary but not sufficient to induce functional improvements (Table A5).

#### 5.2.2. The Influence of Lutein Oral Supplementation on AMD Onset and Progression (Table A6)

The effectiveness of oral supplementation with lutein or other specific nutrients in preventing or delaying AMD onset is not supported by strong scientific evidence from RCTs [48,76].

On the other hand, two prospective large scale RCTs, AREDS [76], published in 2001, and AREDS2 [14], released in 2012, have demonstrated that a daily intake of high doses of vitamins and other antioxidants, although ineffective in preventing AMD onset [48], may significantly decrease the risk of AMD progression from intermediate to advanced AMD stages as compared with placebo [51]. The AREDS2 formula, enriched with lutein and zeaxanthin, was associated with a significantly lower risk of progression to advanced AMD stages and with a reduced risk of GA progression toward the fovea [124].

Some smaller studies have suggested a possible role of dietary lutein supplementation in slowing down AMD progression [109,114,115,116].

Moreover, a recent Cochrane systematic review [51], including 11952 AMD patients investigating the safety and efficacy of antioxidant dietary supplements on reducing AMD progression, has concluded that, although AREDS formulas may likely slow down AMD progression to late stages (low certainty of evidence), supplementation with lutein with or without zeaxanthin may have little or no effect on AMD progression.

#### 5.2.3. The Lutein Oral Supplementation Safety Profile

Lutein oral supplementation has shown a high safety profile. The AREDS2 study, where the assumption of a daily lutein dose of 10 mg for 5 years was required, did not report adverse effects, with the exception of minor skin yellowing [14]. Other studies investigating the effect of lutein supplementation at daily dosages of 20–40 mg for more than 6 months did not find any adverse effects [15,16]. A single case of persistent lutein crystal deposition in the inner retinal layers of the fovea after daily lutein supplementation of 20 mg for 8 years has been published [125]. Based on these data, lutein has been classified by the US Food and Drug Administration (FDA) as Generally Regarded as Safe (GRAS) [126]. Moreover, according to the Council for Responsible Nutrition, lutein is considered to be safe at doses higher than 20 mg/day [127].

However, some theoretical concerns about carotenoid supplementation have been expressed [52]. After capturing ROS and free radicals, macular xanthophylls are oxidized to their corresponding radical cations, which are pro-oxidant, can damage the retina, and must be reduced to regenerate the original carotenoids [128].

#### 5.2.4. The Limitations of the Lutein Oral Supplementation

The most important limitations of lutein oral supplementation as therapeutic strategy in AMD patients include the lack of guidelines about dosages and treatment duration; the need for daily and continuous intake, which may reduce patient compliance [13,15,16,36,55,77]; and variable lutein bioavailability after oral consumption, which is related to several different dietary, metabolic, and genetic factors, inducing significant inter-individual differences in lutein absorbance, transport, and metabolism [8,17,55,56,57].

Moreover, the final retinal concentration of orally consumed lutein is also limited by the presence of complex static and dynamic ocular physiological barriers [129,130]. In particular, the inner and outer retinal/ocular barriers, formed, respectively, by the tight junctions of retinal capillary endothelial cells and of RPE cells, represent the most important obstacle for systemically administered drugs to reach therapeutic concentrations inside the retina [129,130].

## 6. The Trans-Scleral Iontophoresis

The limitations related to conventional lutein oral supplementation have prompted new drug delivery strategies [131,132].

However, implants and intravitreal injections of lutein have been shown to be significantly invasive and expensive [131].

In this scenario, ocular iontophoresis represents a new, non-invasive method to enhance the ocular penetration of specific drugs [131,132], and has recently been used to deliver lutein inside the retina [26,27,28,29,30,31].

### 6.1. Iontophoresis as Drug Delivery System

Iontophoresis is a physical method that uses low-intensity electric currents to deliver ionized or charged molecules from a liquid formulation to a target [18].

Since the early 1990s, iontophoresis has been used as a non-invasive technique to increase the penetration of drugs into human tissues [19], and it has been applied in many different medical fields to treat pain, inflammation, infections, and cancers [20,25,133,134,135].

The standard iontophoretic equipment is composed of a generator of direct or alternating electric currents, a pump to drive the solutions containing the substances that should be delivered, and two electrodes of opposite charge [18]. The optimal molecule to be delivered by iontophoresis is small, hydrophilic, water-soluble, and susceptible to ionization.

Iontophoresis may increase drug penetration into the human body using three different mechanisms [19,20].

- Electrophoresis, or electro-migration, or direct field effect (the Nernst–Planck effect), i.e., the movement of ions caused by the application of an electrical potential gradient. This is the most important transport mechanism for small ionized molecules induced by an electric field.
- Electro-osmosis, which represents the motion of the solvent carrying the molecules induced by an electric potential applied across an ionized membrane, that can increase the movement of ions and neutral molecules. This is the most important mechanism explaining the movement of large monovalent ions and neutral molecules.
- Electro-permeabilization or electroporation, i.e., the alteration of the passive and/or active transport pathway of an anatomical barrier induced by an electric field, can enhance in a reversible or irreversible manner tissue permeability during and after iontophoresis.

Drug penetration and tissue concentration reached by iontophoresis depend on different factors, including electric field force, current density and application time; contact area probe/tissue; size and location of the electrodes; tissue permeability; characteristics of the solution containing the drug; and drug concentration, molecular weight, shape, and surface charge distribution [18,19].

The main advantages of iontophoresis over other conventional drug delivery systems include non-invasiveness; simplicity of use; targeted, faster, higher, and sustained tissue drug concentrations; avoidance of systemic unpleasant side effects; and enhanced patient compliance [20].

Iontophoresis side effects relate to current density and total application time, application site, biological tissues, and the characteristics of the delivered drugs [19,20]. Tissue damage related to iontophoresis application is classified as thermal, chemical, and mechanical, and includes skin or mucosal burns, redness, flogosis, rash, irritation, etc. [136].

General contraindications to iontophoretic treatments are pregnancy, epilepsy, presence of a pacemaker or other implanted electronic devices, metal implants, open wounds, cuts, mucosa interruption, or active infections in the treatment area [136].

### 6.2. Iontophoresis in Ophthalmology

In the last decades, iontophoresis has been used in ophthalmology to deliver various drugs into the eye [21,22,23,24,25].

The basic ocular iontophoresis equipment is composed of a power source for electric current, an ampere meter, a pump and dose controller to drive the drug, a pair of electrodes, and an eye drug applicator, i.e., an eye-cup solution [21,22,23,24,25]. The procedure is preceded by applying a topical anesthetic to the treated eye. During iontophoresis, a low-intensity current applied to an active electrode located on the cornea or on the sclera flows to a passive electrode placed on the periorbital skin, thus inducing the penetration of charged molecules into the ocular tissues [21,22,23,24,25].

Based on the application site, ophthalmic iontophoresis can be divided into trans-corneal iontophoresis, developed to treat corneal and anterior segment pathologies, and trans-scleral iontophoresis, used to deliver drugs into the vitreous and the retina. Due to the higher permeability and larger surface of the sclera as compared to the cornea, the drug penetration rate after trans-scleral iontophoresis is much higher than that after the trans-corneal application [23]; moreover, the negative charge of the sclera increases the anodic delivery of positively charged formulations [23].

#### 6.2.1. Iontophoresis in Ophthalmology: Experimental Studies (Table 1)

Numerous histopathologic and toxicity tests have been performed in animal studies to provide protocols defining the current intensity and duration of the application of ocular iontophoresis and guarantee the safety of the procedure [137,138,139,140,141]. Reported adverse effects of the procedure in animal studies include corneal edema, burns or inflammatory infiltration, reduced endothelial cell count, corneal opacification, scleral stromal edema, retinal layers disorganization, and choroidal and retinal flogosis and/or necrosis [137,138,139,140,141].

**Table 1:** Ocular iontophoresis experimental studies.

**Keratoconus**	Iontophoresis-assisted corneal-cross-linking [C-CXL] has been studied in many animal models, and has been shown to quicken and increase the penetration of riboflavin into the corneal stroma and to be associated with lower epithelial damage/corneal swelling and faster recovery as compared with conventional C-CXL [142].
**Degenerative myopia**	Animal studies in rabbits have demonstrated that an iontophoresis–assisted scleral riboflavin/UVA cross-linking procedure may be effective in delivering riboflavin into the sclera and reducing abnormal scleral elongation, and it could potentially control degenerative myopia progression [143].
**Bacterial ocular infections**	Trans-scleral iontophoresis was able to provide high concentrations of selected antibiotics (cefazolin, ticarcillin, gentamicin, and vancomycin) into the vitreous body, retina, and choroid in a rabbit animal model [144,145,146]. Trans-corneal iontophoretic treatment with antibiotics in rabbit (gentamicin) [147] and porcine animal models (ciprofloxacin) [148] has shown promising results in treating corneal infections.
**Herpes simplex virus (HSV) and cytomegalovirus (CMV) infections**	Trans-scleral iontophoresis was able to deliver effective doses of ganciclovir [149] or foscarnet [150] into the vitreous cavity of rabbit eyes to treat CMV retinopathy. Preliminary studies in porcines have demonstrated that trans-corneal and trans-scleral iontophoresis of acyclovir prodrugs may be useful in the treatment of HSV infections of the eye anterior and posterior segments [151].
**Fungal ocular infections**	Rabbit animal models and ex vivo studies have shown that ocular iontophoresis of ketoconazole [152,153] or caspofungin acetate [154] can provide high drug concentration into the eye.
**Non-infectious eye inflammations**	Trans-corneal and trans-scleral iontophoresis of corticosteroids, such as dexamethasone and triamcinolone acetonide, in animal models (rabbit and porcine) has shown rapid and high concentrations of the drugs inside the anterior and posterior segments of the eye [155,156]. Trans-scleral iontophoresis of acetylsalicylic acid provided rapid and sustained concentrations of the drug inside rabbit eyes, avoiding systemic side effects [157]. As compared to intravitreal injections, trans-scleral iontophoresis of methotrexate in rabbit eyes provided faster and twice-higher scleral, vitreous, and retinal drug concentrations, which may have clinical value in managing ocular inflammatory diseases and intraocular lymphoma [158].
**Inherited retinal degenerations**	Trans-scleral iontophoresis of plasmid DNA containing therapeutic genes in a mouse animal model of autosomal recessive retinitis pigmentosa induced morphological and functional improvements of photoreceptors as evaluated using microscopy and electroretinogram [159].
**Retinoblastoma**	Experimental studies performed in a murine model of retinoblastoma have shown that the trans-scleral iontophoretic delivery of carboplatin allows for drug concentrations in the retina, choroid, vitreous, and optic nerve that are significantly greater than those after intravenous drug administration, avoiding systemic toxicity [160].
**Macular neovascularization (MNV)**	Recent animal studies of CNV have demonstrated that 0.6 mg of bevacizumab can be delivered into a rabbit eye in vivo using a 20-min trans-scleral iontophoretic treatment [161].
**Neurodegenerative ocular pathologies**	A recent study has demonstrated that trans-scleral iontophoresis can efficiently deliver cytochrome c into the eye posterior segment [162].
**Age-related macular degeneration (AMD)**	Trans-scleral iontophoresis has been demonstrated to be able to rapidly and efficiently deliver effective doses of lutein into the retina of human donor eye globes [26,27].

Several experimental animal studies have demonstrated that iontophoresis is able to efficiently, rapidly, and safety deliver many drugs into the anterior (trans-corneal) and posterior (trans-scleral) segments of the eye, including riboflavin [142,143]; numerous antibiotics, such as gentamicin, ciprofloxacin, cefazolin, amikacin, and vancomycin [144,145,146,147,148]; antiviral drugs, such as acyclovir, ganciclovir and foscarnet [149,150,151]; antifungal medication, such as ketoconazole and caspofungin acetate [152,153,154]; corticosteroids, such as dexamethasone and triamcinolone acetonide [155,156]; non-steroidal anti-inflammatory drugs, such as acetylsalicylic acid [157] and methotrexate [158]; plasmids containing therapeutic genes [159]; anti-cancer drugs, such as carboplatin [160]; anti-VEGF molecules, such as bevacizumab [161]; and molecules with anti-apoptotic and regenerative properties, such as cytochrome c [162].

Moreover, recent ex vivo studies on donor human eye globes have demonstrated that trans-scleral iontophoresis is able to rapidly deliver effective doses of lutein into the retina [26,27].

#### 6.2.2. Iontophoresis in Ophthalmology: The Clinical Studies (Table 2)

Numerous histopathologic and toxicity tests have been performed in animal studies to provide protocols defining current intensity and duration of the application of ocular iontophoresis and guarantee the safety of the procedure [137,138,139,140,141].

The application of ocular iontophoresis in clinical practice has been preceded by tolerance tests in healthy volunteers, which demonstrated a high safety profile of the procedure and good patient acceptance [28,163].

**Table 2:** Ocular iontophoresis clinical studies.

**Dry eye disease (DED)**	Trans-corneal iontophoresis of iodide, a reactive oxygen species scavenger (iodide) [164] and of dexamethasone phosphate [165] has been proposed to treat signs and symptoms of DED with promising preliminary results.
**Keratoconus**	Iontophoresis can increase the penetration of riboflavin into the corneal stroma during the riboflavin 0.1%/ultraviolet-A (UVA) corneal cross-linking (C-CXL) procedure used to stop keratoconus progression. Recent clinical studies have demonstrated that, as compared to conventional C-CXL, iontophoresis-assisted C-CXL is associated with faster and higher refractive and visual acuity improvements and lower epithelial damage/corneal swelling [166,167].
**Corneal graft rejection**	A case report showed that trans-scleral iontophoresis of methylprednisolone may be useful in the treatment of severe acute corneal graft rejections [168].
**Non-infectious anterior scleritis**	A recent randomized double-masked study demonstrated the higher efficacy and safety of trans-scleral iontophoresis of dexamethasone phosphate in this pathology as compared to systemic corticosteroid administration [169].
**Non-infectious anterior uveitis**	Previous clinical studies have shown that treatment with dexamethasone phosphate delivered by trans-corneal iontophoresis for treating severe non-infectious anterior uveitis may be more effective and well-tolerated than corticosteroid eyedrops [170,171].
**Diabetic retinopathy**	Some promising preliminary results have been achieved with trans-scleral iontophoresis of insulin [21].
**Uveal melanoma**	Trans-scleral iontophoresis has been proposed to increase the penetration of anti-cancer drugs into uveal melanoma cells [172].
**Age-related macular degeneration (AMD)**	Preliminary clinical studies have demonstrated that trans-scleral iontophoresis is able to safely, rapidly, and efficiently deliver effective doses of lutein into the retina of AMD patients, increasing MPOD levels and preserving BCVA [28,29,30,31].

MPOD = macular pigment optical density; BCVA = best-corrected visual acuity.

Preliminary clinical studies have suggested that ocular iontophoresis may be useful for treating different ocular pathologies by efficiently delivering specific drugs into the eye. Trans-corneal iontophoresis of iodide has shown promising results in reducing the signs and symptoms of dry eye disease [164]; trans-corneal iontophoresis of riboflavin has been demonstrated to improve corneal cross-linking procedure results [165,166]; trans-corneal and trans-scleral iontophoresis of corticosteroids, such as dexamethasone phosphate or methylprednisolone, has provided encouraging results in the treatment of dry eye disease [167], corneal graft rejection [168], non-infectious anterior scleritis [169], and non-infectious anterior uveitis [170,171]; and trans-scleral iontophoresis of insulin and anti-cancer drugs have been proposed to manage diabetes [21] and uveal melanoma [172], respectively.

Finally, recent pilot clinical studies have demonstrated that trans-scleral iontophoresis is able to rapidly and efficiently deliver effective doses of lutein into the retina of AMD patients [28,29,30,31].

#### 6.2.3. Ocular Iontophoresis Safety (Table 3)

Although ocular iontophoresis is considered to be a non-invasive technique, its safety and tolerability strongly depend on the protocol and ophthalmic formulation. The electrical dose is determined by current density and exposure time, both of which are relevant to the risk of tissue injury. Additional factors may affect local exposure and toxicity, including application site, probe/surface contact, drug concentration, pH, and other formulation characteristics [25].

**Table 3:** Ocular iontophoresis adverse effects.

First Author, Year of Publication, Country	Study Type/Follow-Up	Model/Participants	Iontophoresis Type/Device	Iontophoresis Drug Solution/Procedure Characteristics	Reported Adverse Effects
Parkinson TM, 2003, USA [163]	Prospective, non-randomized, uncontrolled single-arm clinical study	24 healthy volunteers/24 eyes	Trans-scleral/OcuPhor trade mark hydrogel drug delivery applicator (ActivaTek, North Coast Medical Inc., USA)	Balanced salt solution/16 subjects received 0 mA and 2 of the following DC currents: 0.1, 0.5, 1.0, 2.0, 3.0, or 4.0 mA for 20 min; 6 subjects received 3 mA for 20 min and 1.5 mA for 40 min	- Exposure to 0–3.0 mA for 20 min or 1.5 mA for 40 min: minimal/mild transient tingling, burning, aching, photopsia, and metallic taste; - exposure at 4.0 mA for 2 min: 2 out of 4 subjects complained of a burning sensation and showed conjunctival fluorescein staining that resolved within 24 h.
Patane MA et al., 2011, USA [165]	Prospective, single-center, double-masked, randomized, comparative clinical study	103 dry eye patients (n = 103)/103 eyes randomized into 1 of 3 treatment groups: 2.5 mA/3 min (n = 41); 3.5 mA/3 min (n = 37); 3.5 mA/3 min (placebo, n = 25)	Trans-scleral/Eyegate(^®^) II ocular iontophoresis delivery system (Eyegate Pharmaceuticals, Inc., Waltham, MA, USA)	Dexamethasone phosphate (40 mg/mL ophthalmic solution, EGP-437)/2.5 mA/3 min (n = 41); 3.5 mA/3 min (n = 37); 3.5 mA/3 min (placebo, n = 25)	A total of 87% of patients experienced at least one adverse event; 199 out of 254 treatment-emergent adverse events (78%) were ocular. Ocular hyperemia was reported in 39.0%, 48.6%, and 44.0% of patients in the low-dose, high-dose, and placebo groups, respectively; punctate keratitis occurred in 14.6%, 16.2%, and 4.0%, respectively. Ocular adverse events resolved without sequelae within 24 h.
Cohen AE, 2012, USA [170]	Prospective, phase I/II, multicenter, double-masked, parallel group, randomized clinical trial/28 days	42 active noninfectious anterior uveitis patients/42 eyes randomized into 1 of 4 treatment groups: 1.6, 4.8, 10.0, or 14.0 mA-min	Trans-scleral/Eyegate(^®^) II ocular iontophoresis delivery system (Eyegate Pharmaceuticals, Inc., Waltham, USA)	Dexamethasone phosphate (40 mg/mL ophthalmic solution, EGP-437)/ 0.4/4 min, 1.2 mA/4 min, 2.5 mA/4 min, 3.5 mA/4 min	A total of 90% of patients experienced at least one adverse event; 104 of the 134 adverse events (78%) were ocular. The most common ocular adverse events were conjunctival hyperemia (16% of all events), punctate keratitis (11%), conjunctival edema (10%), eyelid edema (8.6%), eye pain (6%), and ocular hypertension (4.7%). Ocular adverse events were minimal/mild and resolved within 48 h.
Sheppard J et al., 2020, USA [171]	Prospective, randomized, double-masked, parallel group, non-inferiority clinical trial/28 days	193 active noninfectious anterior uveitis patients/193 eyes randomized to dexamethasone phosphate TSI (days 0 and 7) or daily self-administered prednisolone acetate 1% eye-drops (tapered schedule, days 0–28)	Trans-scleral/Eyegate(^®^) II ocular iontophoresis delivery system (Eyegate Pharmaceuticals, Inc., Waltham, USA)	Dexamethasone phosphate (40 mg/mL ophthalmic solution, EGP-437)/2.5 mA applied for 4 min	≈50% of patients per group experienced mild and transient device- or procedure-related administration-site reaction. IOP elevations ≥ 6 mmHg were recorded in 13 patients in the TSI group and 24 cases in the topical prednisolone group (*p* < 0.01).
O’Neil EC et al., 2018, USA [169]	Prospective, randomized, double-masked, dose-escalating clinical study/56 days	18 noninfectious non-necrotizing anterior scleritis/20 eyes randomized into 3 groups of treatment: 0.4 mA/3 min (lowest dose), 0.8 mA/3 min (middle dose), and 1.5 mA/3 min (highest dose)	Trans-scleral/Eyegate(^®^) II ocular iontophoresis delivery system (Eyegate Pharmaceuticals, Inc., Waltham, USA)	Dexamethasone phosphate (40 mg/mL ophthalmic solution, EGP-437)/0.4 mA/3 min (lowest dose), 0.8 mA/3 min (middle dose), or 1.5 mA/3 min (highest dose)	The most commonly reported adverse events were symptoms of active scleritis: ocular or periorbital pain (n = 7), exacerbation of scleritis (n = 8), and headache (n = 4). A transitory IOP increase ≥ 6 mmHg was registered in 2 cases.
Lombardo et al., 2017, Italy [166]	Prospective randomized controlled comparative clinical trial/12 months	25 progressive keratoconus patients/34 eyes randomized into iontophoresis-assisted trans-epithelial cross-linking (CXL) (22 eyes) or standard CXL (12 eyes)	Trans-corneal/ IONTORETINA (OffHealth SpA, Italy). After iontophoresis, UVA irradiation was performed as in the standard CXL procedure (10 mW/cm^2^ ultraviolet A irradiation for 9 min)	Dextran-free 0.1% riboflavin-5-phosphate solution/1 mA applied for 5 min	Mild, transient grade 0.5 corneal haze occurred in two eyes (10%), which resolved by 6 months. Central corneal thickness and endothelial cell density showed no significant changes at 12 months.
Alqudah N et al., 2025, Qatar, Jordan, Iraq [167]	Retrospective, observational cohort clinical study	107 progressive keratoconus patients/107 eyes that underwent iontophoresis-assisted trans-epithelial CXL (53 eyes) or standard CXL (54 eyes)	Trans-corneal/I-ON XL, SOOFT, Montegiorgio, Italy). After iontophoresis, UVA irradiation was performed as in the standard CXL procedure (cm2 ultraviolet A irradiation for 9 min)	Dextran-free hypo-osmolar riboflavin phosphate solution containing benzalkonium chloride (Ricrolin+, SOOFT, Montegiorgio, Italy)/1 mA applied for 5 min	Postoperative complications were absent in 40% of patients in the standard CXL group and 93% of patients in the iontophoresis group. Mild corneal haze was significantly more frequent in the standard CXL group (54%) than in the iontophoresis group (5.5%) (*p* < 0.001).
Lombardo M et al., 2022, Italy [28]	Prospective, non-randomized, uncontrolled single-arm pilot clinical study/3 months	9 intermediate/late AMD patients/9 eyes	Trans-scleral/IONTORETINA (OffHealth SpA, Italy)	Hypotonic liposomal solution of 0.1% lutein composed of FloraGLO crystalline lutein encapsulated in positively charged liposomes using phospholipon 90H, octadecylamine, and distilled water (LIPO+, OffHealth SpA, Italy)/2.5 mA applied for 4 min	A total of 89% of patients (8 out of 9 cases) complained of mild ocular itching, discomfort, or lacrimation.
Mastropasqua R et al., 2026, Italy [29]	Prospective, non-randomized, uncontrolled single-arm, longitudinal, pilot clinical study/6 months	15 intermediate AMD patients/15 eyes	Trans-scleral/IONTORETINA (OffHealth SpA, Italy)	Hypotonic liposomal solution of 0.1% lutein composed of FloraGLO crystalline lutein encapsulated in positively charged liposomes using phospholipon 90H, octadecylamine, and distilled water (LIPO+, OffHealth SpA, Italy)/2.5 mA applied for 4 min	No adverse events
Rinaldi M et al., 2026, Italy [30]	Prospective, non-randomized, uncontrolled single-arm, longitudinal, pilot clinical study/6 months	30 intermediate AMD patients/30 eyes	Trans-scleral/IONTORETINA (OffHealth SpA, Italy)	Hypotonic liposomal solution of 0.1% lutein composed of FloraGLO crystalline lutein encapsulated in positively charged liposomes using phospholipon 90H, octadecylamine, and distilled water (LIPO+, OffHealth SpA, Italy)/2.5 mA applied for 4 min	No adverse events; stable ODSI score
Rinaldi M et al., 2026, Italy [31]	Prospective, non-randomized, longitudinal, pilot comparative clinical study/6 months	60 consecutive patients/60 eyes. Patients were non-randomly assigned to one of two treatment groups: lutein trans-scleral iontophoresis (30 patients) or daily oral supplementation (10 mg lutein + 2 mg zeaxanthin + other antioxidants) (30 patients)	Trans-scleral/IONTORETINA (OffHealth SpA, Italy)	Hypotonic liposomal solution of 0.1% lutein composed of FloraGLO crystalline lutein encapsulated in positively charged liposomes using phospholipon 90H, octadecylamine, and distilled water (LIPO+, OffHealth SpA, Italy)/2.5 mA applied for 4 min	No adverse events

mA = Milliampere; TSI = Trans-scleral Iontophoresis; IOP = Intraocular Pressure; CXL = Cross-linking; UVA = Ultraviolet A; AMD = Age-related Macular Degeneration; ODSI = Ocular Surface Disease Index.

Reported adverse effects of the procedure in animal studies include corneal edema, burns or inflammatory infiltration, reduced endothelial cell count, corneal opacification, scleral stromal edema, retinal layers disorganization, and choroidal and retinal flogosis and/or necrosis [137,138,139,140,141].

Ocular signs and symptoms complaints after ocular iontophoresis treatment include ocular hyperemia, corneal or conjunctival fluorescein staining, ocular discomfort, corneal or conjunctival edema, vision blurred, eye irritation, eye pain, foreign body sensation, photopsia, photophobia, dry eye, eye pruritis, abnormal eye sensation, eyelid erythema, eyelid margin crusting, and increased lacrimation [163,165,166,167,169,170,171] (Table 3).

Ocular side effects complaints after lutein trans-scleral iontophoresis included mild ocular itching, discomfort, or increased lacrimation [28,29,30,31] (Table 3).

## 7. Lutein Trans-Scleral Iontophoresis in AMD Management

**Figure 1:**
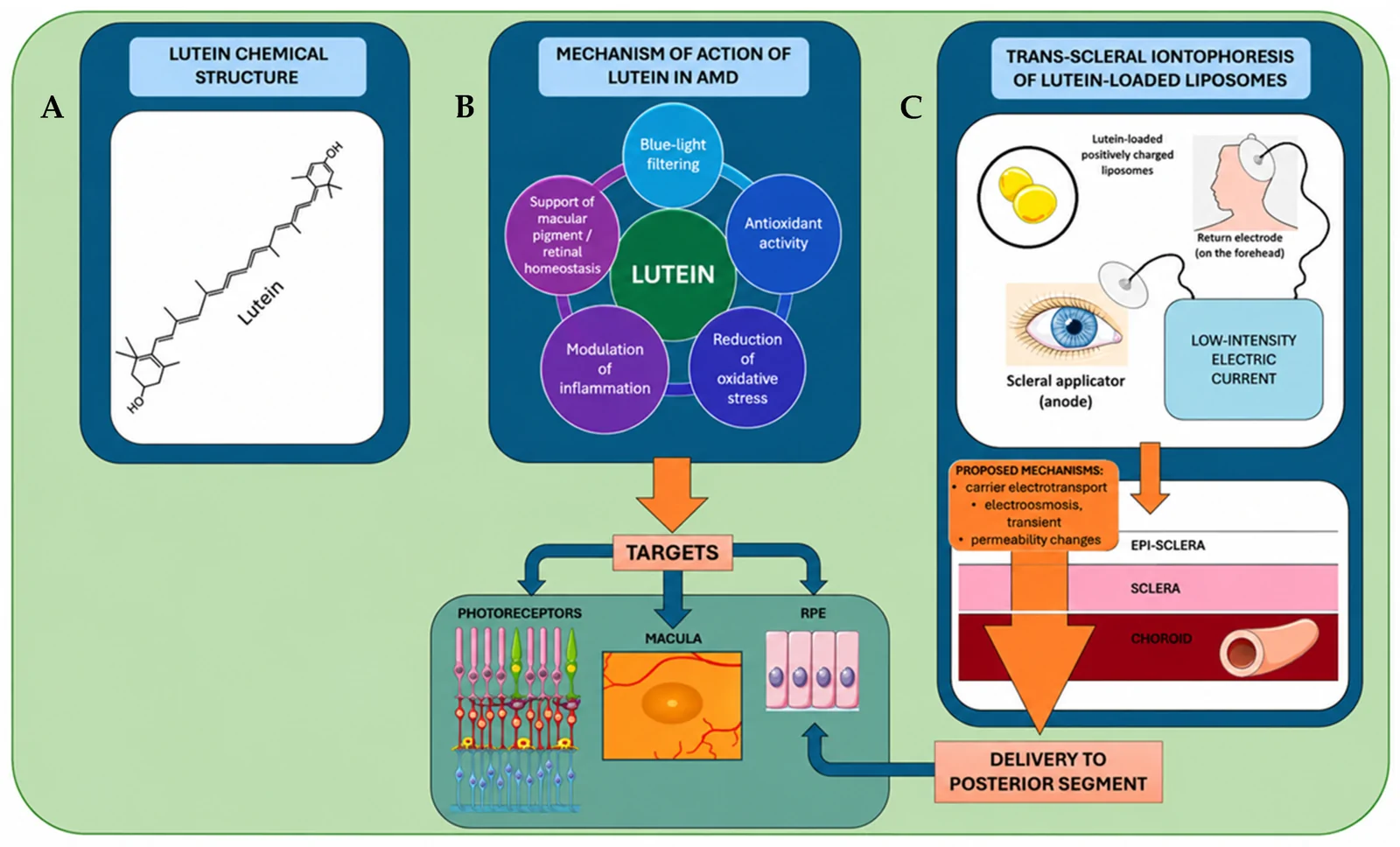
Schematic overview of lutein structure, proposed mechanisms of action in age-related macular degeneration, and trans-scleral iontophoretic delivery to the macula. Panel (**A**) shows the chemical structure of lutein. Panel (**B**) summarizes the main proposed biological actions of lutein in AMD, including blue light filtering, antioxidant activity, reduction of oxidative stress, modulation of inflammatory pathways, and support of macular pigment and retinal homeostasis. Panel (**C**) illustrates the treatment setup and the proposed mechanism of trans-scleral iontophoretic delivery of lutein-loaded positively charged liposomes. A scleral applicator connected to a low-intensity electric current is applied to the ocular surface, while a return electrode is positioned on the forehead. The electric field is thought to enhance delivery mainly through electrotransport of the positively charged carrier system, with possible contributions from electroosmotic flow and transient changes in tissue permeability, thereby promoting drug delivery through scleral tissues toward the posterior segment and macular region. Image(s) provided by Servier Medical Art (https://smart.servier.com, URL accessed on 5 August 2026), licensed under CC BY 4.0 (https://creativecommons.org/licenses/by/4.0/, accessed on 5 August 2026).

Trans-scleral iontophoresis of positively charged lutein ophthalmic formulations represents a new non-invasive strategy to deliver lutein into the retina [26,27,28] [Figure 1].

The rationale of the procedure is to provide targeted, fast, high, and sustained retinal lutein concentrations, increasing lutein retinal bioavailability and therapeutic efficacy, and enhancing patient compliance.

Lutein is an electrically neutral molecule, and its incorporation into positively charged liposomes provides a positive charge to the formulation, enabling its delivery by iontophoresis [26,27]. Iontophoresis is expected to enhance the transport of the positively charged lutein liposomal carriers predominantly through the electro-migration mechanism. Following the deposition within the scleral and choroidal tissues, lutein may be further distributed toward the retina by passive diffusion along concentration gradients [26,27].

Two histologic ex vivo studies [26,27], one preliminary clinical study released in 2022 [28], and three brand-new pilot clinical studies conducted in AMD patients published in 2026 [29,30,31] have shown that trans-scleral iontophoresis of lutein is well-tolerated and effective in enriching the macular pigment of the human eye in a few minutes (Table 4).

All presented ex vivo [26,27] and clinical studies [28,29,30,31] used the same trans-scleral iontophoretic device (IONTORETINA; Offhealth Spa, Florence, Italy) composed of a power supply battery (K-IONO, Offhealth spa, Italy) that was set to deliver current with an intensity of 2.5 mA for 4 min; a negative single-use patch (passive electrode) applied on the periorbital skin (ex vivo studies) or on the patient’s forehead (clinical studies); and a single use scleral applicator (active electrode) positioned on the treated eye. The two electrodes are connected to the power supply unit via cables. The scleral applicator was filled with 2 mL of a hypotonic liposomal solution of 0.1% lutein composed of FloraGLO crystalline lutein (Kemin Food L.C., Des Moines, IA, USA) encapsulated in positively charged liposomes using phospholipon 90H (Lipoid GmbH, Ludwigshafe, Germany), octadecylamine, and distilled water (LIPO+, OffHealth SpA, Italy). A topical anesthesia (0.4% oxybuprocaine, Novesina, Novartis, North Sydney, Australia) was applied before all clinical treatments.

In the first ex vivo experimental study, published in 2016, Sousa–Martin et al. [26] applied trans-scleral iontophoresis of lutein to four donor bank human eyes, and two donor eyes were used as controls, and quantified the amount of lutein reaching the macula by calculating the intensity of the lutein fluorescence band spectrum using a two-photon microscope. One hour after iontophoresis, lutein-enriched liposomes were detected in both central and peripheral retina (but not in the RPE nor in the choroid), concentrated into the macula, located between the inner retina and the outer segments of the photoreceptors, with an estimated increase in macular lutein concentration of 1.9 times in comparison to baseline levels.

In their second ex vivo experimental study, published in 2018, Lombardo et al. [27] used eight donor bank human eye globes and Resonance Raman Spectroscopy to quantify the amount of lutein delivered into the retina by trans-scleral iontophoresis: six donor eye globes underwent lutein trans-scleral iontophoresis, and the other two served as controls. In comparison with controls, forty minutes after treatment, lutein concentration in the six treated eye globes appeared significantly higher in the inner sclera, choroid, and retinal periphery, whereas it did not reach statistical significance in the macula.

In a pilot clinical study [28] (Table 4) published in 2022, lutein trans-scleral iontophoresis was performed on nine AMD patients in order to investigate the safety and tolerability of the procedure. Ocular symptoms reported after treatment included ocular itching, lacrimation, and mild discomfort; however, no major adverse effects were reported, so the authors suggested that lutein trans-scleral iontophoresis may be considered a safe and well-tolerated procedure.

The study by Mastropasqua et al. [29] (Table 4) included 15 patients affected by intermediate-stage AMD. All participants underwent a single session of lutein trans-scleral iontophoresis. BCVA and MPOD were measured using heterochromatic flicker photometry (HPF), and macular spectral domain–optical coherence tomography (SD-OCT) and microperimetry were assessed at baseline and 1, 3, and 6 months after treatment. The most important study findings include a significant MPOD increase at 1 and 3 months, with return to pre-treatment values at 6 months; significant and progressive SD-OCT central macular thickness reduction; significant negative correlation between MPOD increase and microperimetric retinal sensitivity decline; and overall BCVA stability across all visits. No adverse effects were reported. The transient MPOD increase after treatment, lasting approximately 3–4 months, was explained by considering the metabolic turnover of the macular xanthophylls, the limited intra-retinal lutein reservoir [7,8], and the sustained oxidative stress in aging or AMD retinas that may accelerate pigment depletion [103]. The authors recommended, therefore, repeated iontophoresis sessions at approximately 3-month intervals. The central macular thickness reduction was attributed to drusen regression and macular retinal layers remodeling, as previously speculated by other authors [173].

In the study by Rinaldi et al. [30] (Table 4), a single session of lutein trans-scleral iontophoresis was applied to 30 intermediate-stage AMD patients. Pre- and post-treatment visual performances were assessed by evaluating BCVA and MPOD values, measured via one-wavelength fundus reflectometry, and microperimetry parameters. Possible side effects were also monitored. At 6 months, MPOD levels and microperimetry mean sensitivity, mean defect, and fixation stability significantly increased compared with baseline values, whereas BCVA did not show significant changes. No side effects were registered.

The last published study [31] (Table 4) is a prospective, non-randomized pilot study including 60 patients (one eye per patient) affected by intermediate AMD that received either daily oral supplementation with the AREDS2 formula (30 patients) or a single session of lutein trans-scleral iontophoresis (30 patients). BCVA, OCT central macular thickness, and MPOD assessed by one-wavelength fundus reflectometry were measured at baseline and after 6 months. The study results showed that both oral antioxidant supplementation and lutein trans-scleral iontophoresis were associated with similar significant MPOD increases, whereas BCVA and central macular thickness remained stable.

## 8. Discussion

The better understanding of AMD pathophysiologic mechanisms, where photo-oxidative stress seems to play a crucial role [5,6] (Table A1), has suggested that macular carotenoids, because of their blue light filtering and anti-oxidative properties [12,36] (Table A2), may be of fundamental importance in preserving the health and function of the macula, that their reduction in aging or in predisposed individuals may make the retina more vulnerable to light-induced damage, and that their high dietary intake or supplementation may have a therapeutic potential in AMD patients.

Among macular xanthophylls, lutein is thought to play a leading role in preserving retinal health because it is the most abundant carotenoid and the most efficient blue light filter in the human eye [12].

Epidemiological observational studies investigating the relationship between AMD risk and lutein dietary intake, plasma and macular levels (Table A4), although suggesting a beneficial effect of high lutein dietary intake in reducing the risk of AMD onset and progression, have not provided definitive results. However, it is impossible to exactly extrapolate the efficacy of the dietary intake of lutein from that provided by zeaxanthin or other antioxidants commonly associated with lutein in several nutrients.

Clinical intervention studies exploring the role of lutein supplementation in AMD prevention and management (Table A5 and Table A6), although demonstrating its high safety profile [126], have shown highly heterogeneous results, so its role as a therapeutic approach in AMD patients remains controversial.

First, although some previous studies have demonstrated that lutein oral supplementation may significantly enhance lutein plasma and macular concentrations in both healthy subjects and AMD patients, whether or not this may directly translate into visual performance improvements is still under investigation (Table A5). Heterogeneous study results in this field seem to be mainly related to differences in test sensitivity and baseline retinal structural integrity. MPOD level increase due to lutein oral supplementation may better improve some specific visual functions, such as mesopic or scotopic BCVA, contrast sensitivity, and glare and photostress recovery, rather than photopic BCVA, in both healthy and AMD subjects (Table A5). Moreover, visual function improvements secondary to lutein oral supplementation may be better detected using more sensitive and targeted tests, such as microperimetry and multifocal ERG, rather than conventional psychophysical tests, such as BCVA, in both healthy and AMD patients (Table A5). Finally, MPOD enhancements in AMD patients may translate into better visual performance only in the presence of a partially preserved retinal structural integrity, i.e., before the occurrence of advanced irreversible morphological macular damage, so that lutein oral supplementation may be effective in preserving/improving visual function only in early and intermediate AMD stages, where retinal structural integrity is partially preserved.

Secondarily, large-scale RCTs have demonstrated that a daily oral supplementation with antioxidants, i.e., the AREDS formulas, although not effective in preventing AMD onset [48], may significantly decrease the risk of AMD progression from intermediate to late stages [51,76], and that the addition of macular carotenoids to the original AREDS formula may significantly increase its efficacy [14] (Table A6). AREDS formula oral supplementation has been shown to provide higher benefits in patients having a greater risk of disease progression, i.e., those affected by intermediate AMD or early AMD in one eye and advanced AMD in the other eye. Conversely, it seems to be ineffective in patients with early AMD in both eyes, in the general population without AMD signs, or in subjects with high risk of AMD onset, i.e., subjects with no signs of AMD but a positive family history for AMD [48,51].

In addition to AREDS RCTs, some other limited studies [109,114,115,116] have suggested that a dietary supplementation with lutein ± other antioxidants may be effective in delaying AMD progression (Table A6). A recent large Cochrane systematic review [51] including 11952 AMD patients and investigating the safety and efficacy of antioxidant dietary supplements in slowing down AMD progression has concluded that the efficacy of AREDS formulas has low certainty of evidence and that oral supplementation with lutein with or without zeaxanthin may have little or no effect on AMD progression.

In summary, although the entire issue is still under investigation and widely debated, data from the literature support a possible role of high dietary intake/supplementation of lutein in improving visual performance in AMD patients (Table A5) or limiting AMD progression (Table A6).

Nevertheless, lutein oral supplementation as a therapeutic strategy in AMD patients has two important limitations: the need for daily intake for long periods, which may reduce patient compliance [13,15,16,36,55,77], and the variable lutein bioavailability after oral consumption, which may deeply influence lutein retinal delivery [17].

Aiming to overcome lutein oral consumption limitations and to achieve faster, higher, and sustained lutein concentrations in the retina, new drug delivery systems have been proposed [131,132]. However, implants and intravitreal injections of lutein have been shown to be significantly invasive and expensive [131]. Conversely, trans-scleral iontophoresis of positively charged lutein ophthalmic formulation represents a new non-invasive strategy to deliver lutein into the retina [26,27,28].

Iontophoresis is a physical method that uses low-intensity electric currents to transport ionized or charged molecules to a target [18], and it has been applied in many different medical fields as a non-invasive technique to increase the penetration of specific drugs into human tissues [19,20], including the eye [21,22,23,24,25] (Table 1 and Table 2).

In general, the theoretical advantages of ophthalmic iontophoresis over other ocular drug delivery strategies include [21,22,23,24,25] higher delivery efficacy and drug bioavailability, considering that intraocular drug concentrations reached by iontophoresis appear to be significantly higher and faster than those obtained after systemic administration or after topical eye-drop application into the conjunctival sac; higher safety profile, since iontophoresis requires reduced drug dosages and is associated with lower local and systemic side effects as compared to systemic administration, intravitreal injections, or surgical implants; and higher patient compliance, which is related to the non-invasiveness and the relatively long-lasting results of the method.

On the other hand, ophthalmic iontophoresis may have some drawbacks, which include possible local chemical, mechanical, or thermal side effects related to current density, application time and site, and delivered drug characteristics [19,20,137,138,139,140,141], and lack of high sustained half-life, with the requirement of repeated administrations [25,132].

Moreover, clinical studies have shown that ocular iontophoresis may be considered a well-tolerated procedure, being associated with no or mild and transient ocular adverse signs and symptoms (Table 3).

Trans-scleral iontophoresis of lutein has been recently applied in AMD patients aiming to increase macular pigment levels [28,29,30,31] (Table 4).

Lutein is an electrically neutral molecule, and it needs to be incorporated into positively charged liposomes in order to be delivered by iontophoresis. However, liposome diameter, surface charge, encapsulation efficiency, pH, osmolarity, stability, and sterility validation of the ophthalmic solution were not reported by the currently available ex vivo and in vivo studies [26,27,28,29,30,31].

Based on current evidence, iontophoresis is expected to enhance the transport of the positively charged liposomal carriers of lutein predominantly through the electro-migration mechanism, whereas electro-osmotic solvent flow may additionally contribute to trans-scleral transport [26,27]. Although the procedure might theoretically lead to temporary changes in ocular tissue permeability, a process of electro-poretion or increased permeability of the retinal/blood barriers has not been directly demonstrated. However, the quantitative contributions of electro-migration, electro-osmosis, passive diffusion, and permeability changes to the lutein trans-scleral iontophoresis are still unknown [26,27,28]. Furthermore, the available studies did not determine whether intact liposomes crossed all ocular barriers, where lutein is released from the carrier, or whether it remains encapsulated after reaching the retina. Data from an ex vivo study stated that two-photon excitation fluorescence imaging performed 1 h after the procedure revealed retinal particles measuring ≥5 μm with fluorescence characteristics consistent with lutein-enriched liposomes [26]. The size of these retinal particles cannot be interpreted as the original liposome diameter, and neither method assessed the fraction of the applied dose reaching the retina or directly demonstrated whether lutein remained encapsulated after trans-scleral delivery [26,27]. Moreover, the available studies estimated lutein retinal concentrations after the procedure rather than the proportion of the administered dose of lutein reaching the retina, so the efficiency of the procedure is still undetermined.

Previous ex vivo experimental studies on eye donor bank eyes [26,27] (Table A2) have demonstrated that trans-scleral iontophoresis of lutein may represent an innovative, non-invasive method to provide a more localized, rapid, and sustained increase in lutein retinal concentrations, thus overcoming the main limitations associated with lutein oral supplementation. Nevertheless, these studies use different MPOD measurement techniques and provide different data regarding iontophoretic lutein macular enrichment. Moreover, although ex vivo studies are useful for demonstrating the biological feasibility of the procedure, their clinical relevance is limited by several factors, including small sample size, post-mortem tissue changes, absence of blood flow and physiological clearance, and uncertainty regarding lutein cellular localization.

More recently, preliminary clinical studies on AMD patients [28,29,30,31] (Table 4) have shown that lutein trans-scleral iontophoresis is an effective, non-invasive and well-tolerated method to deliver lutein into the human retina in vivo [28], suggesting its possible use as a new AMD therapeutic approach.

Lutein trans-scleral iontophoresis in intermediate AMD patients has indeed provided significant increases in MPOD levels [28,29,30,31] (Table 4). However, MPOD baseline values and percentages of MPOD enhancement during the follow-up were significantly different among studies [29,30,31] (Table 4). The study of Mastropasqua et al. [29] found the highest MPOD increase 1-month post-treatment (+9.4%), with a return to baseline values after 6 months. On the contrary, the studies of Rinaldi et al. [30,31] found significant but dissimilar MPOD increases 6 months post-treatment (+6.5% and +22.5%, respectively). Differences in study population, MPOD measurement methods and their test/retest variability, whose influence may be particularly significant in small cohort studies, may partially account for these heterogeneous results.

In addition, the enhanced MPOD provided by the procedure was shown to be transient, suggesting the need for repeated sessions at approximately 3-month intervals in order to maintain effective retinal lutein concentration [29].

However, as widely discussed above, an in vivo increase in MPOD represents an indirect marker of macular carotenoid content, but its automatic translation into morphological and/or functional benefits or delaying of AMD progression is still under investigation (Table A5 and Table A6). Moreover, the equivalence between one lutein trans-scleral iontophoresis session and 6 months of daily oral lutein supplementation, as suggested by Rinaldi et al. [31], can be speculated on but not clearly demonstrated by a small, short-term, non-randomized, pilot clinical study.

Lutein trans-scleral iontophoresis has been associated with preserved BCVA during a 3/6-month follow-up [28,29,30,31] (Table 4). Nevertheless, in the absence of an untreated or sham control group, BCVA stability over a short follow-up period cannot be attributed to iontophoretic treatment with sufficient certainty of evidence.

The procedure has been linked with some retinal morphologic and functional improvements [29,30,31] (Table 4). However, microperimetric parameter changes linked to the procedure and found in different studies are contradictory [29,30] (Table 4), and the negative relationship between MPOD increase and microperimetric retinal sensitivity found by Mastropasqua et al. [29] represents a potentially unfavorable finding that requires further investigation. Moreover, the OCT central macular thickness reduction found by Mastropasqua et al. [29] (Table 4) and attributed by the authors to drusen regression and retinal remodeling processes may have other possible less favorable explanations, including measurement inter-session variability, OCT segmentation differences, or retinal tissue loss.

Considering procedure safety, no major short-term adverse events have been reported in small patient cohorts and limited follow-up studies [28,29,30,31], and only mild ocular itching, lacrimation, and discomfort have been reported after treatment [31]. However, these findings support short-term feasibility but are insufficient to establish cumulative or long-term safety of the procedure.

Finally, these pilot clinical studies have important limitations [28,29,30,31] (Table 4), including a small participant cohort, lack of precise AMD phenotype definition, short follow-up (3/6 months) [28,29,30,31], subjective [29] or operator-dependent [30,31] methods used to assess MPOD levels, and lack of a sham or control group [28,29,30].

## 9. Conclusions

Trans-scleral iontophoresis of a charged liposomal lutein formulation represents a promising new non-invasive method for increasing macular pigment in patients with intermediate AMD. Preliminary clinical studies have shown that the procedure is easy to use, rapid, well-tolerated, and provides a fast and relatively sustained MPOD increase, although it may likely require repeated sessions at approximately 3-month intervals in order to maintain effective MPOD levels. However, since increased MPOD does not automatically imply retinal morphological/functional improvements, the available evidence does not yet demonstrate a clinically meaningful stabilization/improvement in visual function or AMD progression prevention linked to the procedure. Furthermore, equivalence to oral AREDS2 supplementation, speculated by previous authors [31], requires further investigations and, although no major short-term adverse events have been reported in small patient cohorts and limited follow-up studies, the cumulative and long-term safety of the procedure needs to be established.

In order to regard lutein trans-scleral iontophoresis as a pharmacological therapy in AMD patients in the future, several points must be better elucidated, including dosages, cumulative electrical exposure and administration intervals for different races, ages, genders and disease type and severity; exclusion criteria; interaction with other drugs or supplements; applicability of the procedure in eyes with previous surgery or coexisting diseases; procedure reproducibility; long-term and repeated treatment safety; possible inflammatory or allergic reaction to the charged liposomal lutein formulation; retinal, RPE, choroidal, and scleral toxicity; identification of genetic profiles or systemic and/or retinal biomarkers that may be associated with a more effective response to the treatment; efficacy of the treatment in preventing or slowing disease progression, especially in early AMD stages and younger subjects as “primary prevention” to stop the disease before it reaches advanced severe stages.

The administration of functional tests able to detect with high sensitivity subtle differences in visual performance, such as microperimetry or multifocal ERG, and the introduction of new adaptive optic retinal imaging or advanced AI-supplemented imaging techniques in clinics will hopefully provide more detailed retinal imaging/functional data, which may help select subjects who may be the best candidates for lutein trans-scleral iontophoresis or better understand the effects of the procedure on the retina microstructures in real time in vivo. In conclusion, larger randomized, masked, and sham-controlled trials with standardized functional and structural outcomes are required before this approach can be recommended for routine clinical practice.

## Figures and Tables

**Table 4:** Characteristics of the clinical studies investigating efficacy and safety of lutein trans-scleral iontophoresis in AMD patients.

First Author, Year of Publication, Country	Lombardo M et al., 2022, Italy [28]	Mastropasqua R et al., 2026, Italy [29]	Rinaldi M et al., 2026, Italy [30]	Rinaldi M et al., 2026, Italy [31]
**Study design**	Prospective, non-randomized, uncontrolled pilot clinical study	Prospective, non-randomized, uncontrolled single-arm, longitudinal, pilot clinical study	Prospective, non-randomized, uncontrolled single-arm, longitudinal, pilot clinical study	Prospective, non-randomized, longitudinal, pilot comparative clinical study
**N° of patients/N° of eyes**	9 patients/9 eyes	15 patients/15 eyes	30 patients/30 eyes (eye with lower BCVA in case of bilateral eligibility)	60 consecutive patients/60 eyes. Patients were non-randomly assigned to one of two treatment groups: daily oral supplementation (OS group) (10 mg lutein + 2 mg zeaxanthin + other antioxidants) (30 patients) or lutein trans-scleral iontophoresis (LTI group) (30 patients)
**Inclusion criteria**	Intermediate or late wet AMD in either eye, age > 40 years, BCVA ≤ 0.1 logMAR	Intermediate AMD in one or both eyes, age > 55 years	Intermediate AMD in one or both eyes, age ≥ 55 years, manifest refractive refraction between +4 and -4 diopters, BCVA ≥ 80 ETDRS, IOP < 21 mmHg, limited dietary intake of lutein/zeaxanthin	Intermediate AMD in one or both eyes, age ≥ 55 years, BCVA ≥ 0.52 logMAR
**Exclusion criteria**	Late dry AMD, pregnancy, heavy smokers (>20 cigarettes/day), eye diseases other than AMD	Presence of systemic or ocular diseases other than AMD, aphakia, pseudophakia, different AMD stages, oral supplementation with lutein or zeaxanthin or other ocular supplements within the previous 3 months, pregnancy, breastfeeding, heavy smokers (>20 cigarettes/day)	Ocular surgery within the previous 3 months, present or past eye diseases other than AMD, different AMD stages, use of medications known to affect ocular health, oral supplementation with lutein or zeaxanthin or other ocular supplements within the previous 4 weeks, pregnancy, breastfeeding, type 1 diabetes, history of cerebrovascular accidents, uncontrolled hypertension or cardiovascular diseases, heavy smokers (>20 cigarettes/day), high alcohol consumption (>2 drinks/day), hormone replacement therapy, hypersensitivity to components of the medical device	Ocular surgery within the previous 6 months, present or past eye diseases other than AMD, different AMD stages, use of medications known to affect ocular health, oral supplementation with lutein or zeaxanthin or other ocular supplements within the previous 4 weeks, pregnancy, breastfeeding, type 1 diabetes, history of cerebrovascular accidents, uncontrolled hypertension or cardiovascular diseases, heavy smokers (>20 cigarettes/day), high alcohol consumption (>2 drinks/day), hormone replacement therapy, hypersensitivity to components of the medical device
**AMD stage**	Intermediate, late wet	Intermediate	Intermediate	Intermediate
**Device/lutein formulation**	IONTORETINA (OffHealth SpA, Italy)/hypotonic liposomal solution of 0.1% lutein composed of FloraGLO crystalline lutein encapsulated in positively charged liposomes using phospholipon 90H, octadecylamine, and distilled water (LIPO+, OffHealth SpA, Italy)	IONTORETINA (OffHealth SpA, Italy)/hypotonic liposomal solution of 0.1% lutein composed of FloraGLO crystalline lutein encapsulated in positively charged liposomes using phospholipon 90H, octadecylamine, and distilled water (LIPO+, OffHealth SpA, Italy)	IONTORETINA (OffHealth SpA, Italy)/hypotonic liposomal solution of 0.1% lutein composed of FloraGLO crystalline lutein encapsulated in positively charged liposomes using phospholipon 90H, octadecylamine, and distilled water (LIPO+, OffHealth SpA, Italy)	IONTORETINA (OffHealth SpA, Italy)/hypotonic liposomal solution of 0.1% lutein composed of FloraGLO crystalline lutein encapsulated in positively charged liposomes using phospholipon 90H, octadecylamine and, distilled water (LIPO+, OffHealth SpA, Italy)
**Current intensity/treatment duration**	2.5 mA/4 min	2.5 mA/4 min	2.5 mA/4 min	2.5 mA/4 min
**Control**	None		None	Oral supplementation group
**Tests**	BCVA evaluation, OCT imaging, questionnaire investigating tolerability of the procedure	BCVA evaluation, OCT imaging, microperimetry test, MPOD measurement	BCVA evaluation, IOP measurement, anterior segment and fundus examination, EDI-OCT imaging, microperimetry test, MPOD measurement, OSDI questionnaire	BCVA evaluation, IOP measurement, anterior segment and fundus examination, EDI-OCT imaging, MPOD measurement
**Primary outcomes**	Procedure safety (determined by assessing BCVA and OCT-CRT) and tolerability (assessed using a questionnaire)	MPOD changes at all follow-up points	BCVA changes from baseline to 6 months post-treatment	MPOD changes from baseline to 6 months post-treatment
**Secondary outcomes**	None	MPOD changes impact on OCT- CMT, OCT-CVI, and MMS	MPOD, microperimetric parameters and ocular surface changes Post-treatment complications	BCVA and OCT-CMT changes, treatment safety, and tolerability
**MPOD measurement method**	n.a.	Heterochromatic flicker photometry (MP Screener II macular densitometer, MPSII Elektron Technology, Cambridge; United Kingdom)	One-wavelength fundus reflectance method (Visucam 200 system, Zeiss Meditec, Jena, Germany)	One-wavelength fundus reflectance method (Visucam 200 system, Zeiss Meditec, Jena, Germany)
**Follow-up controls**	1 week, 1 and 3 months after treatment	1, 3, and 6 months after treatment	1 day, 1 week, 1 month, and 3 months after treatment to monitor adverse events and 6 months after treatment for repeat tests	6 months after treatment
**Results**	BCVA (logMAR) significantly improved from 0.20 ± 0.2 to 0.11 ± 0.2 the first week post-treatment (*p* = 0.01), then returned to pre-op values. OCT-CRT did not change. The procedure was well-tolerated; only mild ocular itching, discomfort, and lacrimation were reported by 8 of 9 patients	Baseline/1 month/3 months/6 months post-treatment: - MPOD: 0.64 ± 0.2/0.70 ± 0.2/0.65 ± 0.3/0.57 ± 0.2 (significantly higher at 1 and 3 as compared with 6 months post treatment, *p* = 0.01); - BCVA (logMAR): 0.19 ± 0.1/0.19 ± 0.1/0.19 ± 0.10/19 ± 0.1 (stable, *p* > 0.05); - OCT-CMT (µm): 273.2 ± 28/270.9 ± 28/270.9 ± 29/230.2 ± 23 (significantly lower 6 months post-op, *p* ≤ 0.002); - OCT-CVI: 0.67 ± 0.03/0.65 ± 0.03/0.65 ± 0.02/0.65 ± 0.01 (significantly lower at 3 and 6 months post-treatment, *p* = 0.02); - MRS (dB): 22.6 ± 2.9/21.8 ± 3.1/21.6 ± 2.9/22.1 ± 3.3 (significantly lower at 1 and 3 months post-treatment, *p* = 0.03); - Significant negative correlation between MPOD and MRS changes at 3 months post-treatment (*p* = 0.035)	Baseline/6 months post-treatment - Mean BCVA (ETDRS letters): 46.4 ± 18.1 letters/47.8 ± 17.6 (stable, *p* > 0.05); - mean MPOD: 0.17 ± 0.1/0.18 ± 0.1 (significant increase, *p* = 0.03); - MMS (dB): 7.26 ± 5.1/8.18 ± 4.6 (significant increase, *p* = 0.02); - MMD (dB): −11.5 ± 4.9/−10.3 ± 3.8 (significant increase, *p* = 0.02); - ODSI score: 24.1 ± 16.3/24.1 ± 17.5 (stable, *p* > 0.05)	Baseline/6 months post-treatment in LTI and OS groups: - MPOD: 0.40 ± 0.1/0.49 ± 0.1 in LTI group (significant increase, *p* < 0.001); 0.41 ± 0.1/0.47 ± 0.1 in OS group (significant increase, *p* = 0.003); no inter-groups differences (*p* > 0.05); - BCVA (logMAR): 0.17 ± 0.1/0.2 ± 0.1 in LTI group (stable, *p* > 0.05); 0.18 ± 0.1/0.17 ± 0.1 (stable, *p* > 0.05); no inter-group differences (*p* > 0.05); OCT-CMT (µm): 279 ± 24/284 ± 25 in the LTI group (stable, *p* > 0.05); 276 ± 21/281 ± 23 (stable, *p* > 0.05). No inter-group differences (*p* > 0.05)
**Adverse events**	None	None	None	None
**Limitations**	Non-randomized study design, small patient cohort, short follow-up, lack of sham or control group and MPOD evaluation	Non-randomized study design, small patient cohort, short follow-up, lack of sham or control group, subjective MPOD evaluation method	Non-randomized study design, small patient cohort, short follow-up, lack of control group, operator-dependent MPOD measurement method	Non-randomized study design, small patient cohort, short follow-up, operator-dependent MPOD measurement method
**Fundings/Manufacturer involvement**	OffHeathy SpA provided the medical devices for the procedure	None	None	None

AMD = Age-related Macular Degeneration; BCVA = Best-Corrected Visual Acuity; ETDRS = Early Treatment Diabetic Retinopathy Study; IOP = Intraocular Pressure; logMAR = logarithm of the Minimum Angle of Resolution; mA = milliAmpere; OCT = Optical Coherence Tomography; MPOD = Macular Pigment Optical Density; EDI-OCT = Enhanced Depth Imaging-Optical Coherence Tomography; OSDI = Ocular Surface Disease Index; CRT = Central Retinal Thickness; CMT = Central Macular Thickness; CVI = Choroidal Vascularity Index; MMS = Microperimetric Mean Sensitivity; MMD = Microperimetric Mean Defect; n.a. = not applicable.

## Data Availability

No new data were created or analyzed in this study. Data sharing is not applicable to this article.

## Appendix A

**Table A1:** Pathophysiologic pathways involved in AMD onset and progression.

**Light-induced oxidative damage of the complex photoreceptors/RPE/choriocapillaris [blue light hazard] [35]**	Light-induced oxidative damage is due to high-energy lights, such as blue light, that can induce leakage of electrons from the mitochondrial electron transport chain, with ROS formation. ROS act as free radicals, i.e., molecules containing one or more unpaired electrons, having high chemical reactivity. Their oxidation of proteins, DNA, and cellular membrane lipids leads to mitochondrial damage, which increases ROS production, inflammation, and cellular damage. Reasons explaining the high retina susceptibility to photo-oxidative stress include the following: - Constant light exposure: Photoreceptors are responsible for the transduction of light photons into electrochemical signals and are continuously exposed to light. - Highly oxygenated environment: The retina is the highest oxygen-consuming tissue of the human body. Photoreceptor inner segments, site of the light/electrochemical transduction, and RPE cells have high metabolic activity, require great amounts of oxygen, and contain a high number of mitochondria that represent the major site of ROS generation and are, therefore, highly sensitive to oxidative damage. - High polyunsaturated fatty acid content: Photoreceptors’ outer segments are rich in polyunsaturated fatty acids that are particularly prone to ROS peroxidation, with formation of reactive aldehydes that can amplify flogosis and cellular damage.
**Macular pigment depletion [12,36]**	Macular pigment, composed of dietary-introduced carotenoids, is able to filter blue light, preventing ROS formation and, in addition, to act as a scavenger of ROS and other free radicals. Aging processes or metabolic alterations in genetically predisposed subjects may decrease bioavailability and macular storage of dietary carotenoids, thus increasing retinal vulnerability to oxidative damage.
**Inflammatory damage [37]**	Accumulation of ROS and other free radicals into the retina may upregulate several pro-inflammatory cytokines and stimulate the migration of immunitary cells, such as the microglia, leading to inflammation and blood/retinal barrier and choriocapillaris breakdown, which creates a vicious circle of flogosis and cellular dysfunction and death.
**Age-related damage amplification [38]**	Aging increases ROS production due to electron leakage from the mitochondrial electron transport chain, and this is combined with a physiological reduction in the cellular antioxidant capacity. The imbalance between ROS and cellular antioxidant defenses, with ROS accumulation, induces oxidative damage, inflammation, cell dysfunction, and loss. Moreover, the physiological age-related macular pigment reduction may increase retinal vulnerability to light-induced oxidative damage.
**Genetic predisposition [39]**	Several genetic variants have been associated with increased risk of AMD onset and progression and poorer response to treatment. The most important ones are mutations of genes related to the complement system, such as the complement factor H (CFH) and the AMD susceptibility gene 2 (ARMS2), to lipid metabolism and to angiogenic pathways (vascular endothelial growth factor and fibroblast growth factor). Polymorphisms in CHF and ARMS2 alleles together account for a 45% risk of AMD development.
**RPE as weak link of the chain [40]**	RPE is essential for photoreceptors’ function and survival because it is deputed to the phagocytosis of the released photoreceptors’ outer segments. RPE activity impairment due to light-induced oxidative damage leads to accumulation of metabolic by-products, i.e., lipofuscin and drusen. The deposition of metabolic waste creates a physical barrier between photoreceptors and choriocapillaris that reduces the exchange of nutrients, oxygen, and waste products; moreover, it may stimulate chronic inflammation with photoreceptor and RPE damage, and blood/retinal barrier and choriocapillaris breakdown, in a vicious circle of flogosis and cellular damage.

AMD = age-related macular degeneration; RPE = retinal pigment epithelium; ROS = reactive oxygen species.

**Table A2:** Macular carotenoids properties and function.

**Blue light filtering effect**	High-energy visible blue light, having a wavelength between 440 and 490 nm, is highly reactive and may induce free radicals formation and consequent oxidative damage to the eye tissues, resulting in cataract and AMD development [35]. Blue light sources, including computers, smartphones, digital tablets, and light-emitting diode lamps, are extremely diffused, so that eyes are potentially constantly exposed to the risk of blue light-induced oxidative damage. Macular xanthophylls, having a wavelength absorption range between 350 and 540 nm [8,9], can filter 40–90% of the incident harmful blue light depending on concentration, thus protecting the retina against the blue light-induced oxidative stress [9,12].
**Anti-oxidative**	Thanks to their chemical structure, characterized by nine double bonds and two hydroxyl groups located on both sides of the molecule, macular carotenoids are able to function as free radicals scavengers by giving or absorbing free electrons, thus protecting the retina from oxidative damage [7,8,9]; moreover, they may upregulate other antioxidants, such as superoxide dismutase enzymes and glutathione [9].
**Anti-inflammatory**	Macular xanthophylls are able to inactivate ROS and other free radicals that can trigger uncontrolled inflammatory pathways [9,12]; they may downregulate gene expression of several pro-inflammatory molecules, including pro-inflammatory cytokines and pro-inflammatory hormones [9,12], and they may suppress macrophage recruitment [9,12].
**Anti-angiogenic**	Lutein is able to decrease VEGF expression [13,36,55], which is involved in several retinal diseases, including wet AMD.
**Anti-apoptotic, anti-aging and neuroprotective**	Lutein has been shown to reduce cellular damage after retinal ischemia/reperfusion injury, which seems to be related to its anti-inflammatory properties [13,36,55]. Epidemiological studies have found that higher lutein intake was associated with lower levels of aging-related biomarkers [60]
**Cognitive functions modulation**	Macular carotenoids seem to modulate several brain areas associated with visual signal perception and processing, cognition, motor-coordination. and decision-making [9]. Supplementation with lutein and zeaxanthin in healthy adults has been associated with improvements in reasoning skills, complex attention tasks, memory, learning, spatial, visual, and verbal memory [58,59].
**Central visual function optimization**	Likely due to their blue light filtration effect and cerebral visual inputs modulation [9,12], macular carotenoids have been associated with increased central visual acuity and contrast sensitivity and reduced glare, light-scattering, chromatic aberrations, and photo-stress recovery time [8,9,12,36,55].
**Miscellanea**	Maintenance of the structural and functional integrity of the cellular membrane by ROS scavenger activity and by cytoskeleton microtubules binding; promotion of intercellular communication through gap junctions due to their chemical structure; stimulation of the immune system; regulation of the cell cycle; regulation of growth factors; fighting viruses; inhibition of cancer cell growth [7,8,9,12,36,55].

AMD = age-related macular degeneration; ROS = reactive oxygen species; VEGF = vascular endothelial growth factor.

**Table A3:** Macular pigment optical density (MPOD) measurement techniques.

**Psychophysical techniques**	**Heterochromatic Flicker Photometry (HFP) or Color Matching Test [61,62]:** This is the most widely used psychophysical technique. The test involves the presentation of a flickering stimulus composed of two lights with different wavelengths. By varying the intensity of one light, the point at which the flickering disappears indicates macular pigment absorption. The test is non-invasive and simple to perform; however, it requires patient collaboration and may be influenced by individual visual and flicker perception. Moreover, it estimates an overall pigment density, but does not provide information about pigment distribution.
**Indirect systemic measurement techniques**	**Veggie Meter:** This uses reflection spectroscopy to measure the skin level of carotenoids, primarily composed of lycopene, that have a weak correlation with MPOD, but it is not a direct MPOD measure [61].
	**High-performance Liquid Chromatography:** This is an expensive and time-consuming method considered the gold standard to measure the serum carotenoid level that correlates with MPOD, but is not a direct MPOD measure [61].
**Objective techniques**	**Fundus Autofluorescence (FAF)**: This is a non-invasive method that captures and measures fundus autofluorescent emitted light. FAF provides an MPOD estimation based on the ability of the macular pigment to attenuate the fundus background autofluorescence [61].
	**Macular Pigment Reflectometry (MPR) [63]:** This is a non-invasive, accurate, objective method that measures and compares the light reflected from part of the retina with that reflected from the fovea and uses the difference to calculate MPOD. Lights of different wavelengths are used to separately quantify lutein and zeaxanthin optical densities in vivo and their distribution in the different retinal regions.
	**Resonance Raman Spectroscopy (RRS) [64]:** This is a non-invasive, accurate, objective, highly molecular-specific and sensitive method providing a separate measure of lutein and zeaxanthin optical density together with a bidimensional map of their distribution in the human retina in vivo. It is based on the excitation of the retina with a blue laser source that induces the carotenoids to resonate and emit scattered light with different frequencies that are captured and measured.

**Table A4:** Relationship between dietary intake of specific nutrients, lutein plasma, or macular levels (MPOD) and AMD risk.

Nutrients (High Dietary Intake)	Effects
Mediterranean diet, i.e., high consumption of vegetables, fruits, legumes, olive oil, fish, whole grain, nuts, moderate intake of red wine and low intake of red meat	Lower risk of AMD development [45]. Lower risk of AMD progression, especially among smokers [45].
DHA and/or EPA	Lower risk of AMD development [81]. Lower risk of AMD progression [79,80].
β-carotene, calcium, lycopene, zinc, copper, magnesium, folate, vitamin A, vitamin B3, vitamin B6, vitamin C	Lower risk of AMD progression [79,80].
Oleic acid, linoleic acid, mono-unsaturated or saturated fatty acids	Higher risk of AMD progression [79].
Alcohol	Higher risk of AMD development [78]. Lower risk of AMD progression [79].
Total fatty acids, saturated fatty acids, mono-unsaturated fatty acids, poly-unsaturated fatty acids, alpha-linoleic acids	No association with AMD onset [81].
α-carotene, β-cryptoxanthin, iron, zinc, lactose, vitamin B1, vitamin B2, vitamin B12, vitamin E, arachidonic acid, cholesterol, α-linoleic acid, vegetables	No association with AMD progression [79,80].
Lutein ± zeaxanthin ± other anti-oxidants	Lower risk of AMD onset [10,82,83,84,85,86,87] also in subjects with high genetic risk of AMD [84]. Lower risk of AMD progression [11,86] also in subjects with high genetic risk of AMD [11]. No association with AMD onset or progression [88,89,90].
Lutein serum levels	Inverse relationship between serum lutein levels and risk of AMD onset [91,92,93,94] or AMD progression [95,96]. No significant association between lutein serum levels and AMD [88,95,97].
MPOD	Significantly lower MPOD levels in AMD patients [98,99,100,101] or in AMD patients’ relatives [102] than in healthy subjects. No significant difference between AMD and healthy patients [104,105,106]. Significantly higher MPOD levels in AMD patients than in healthy subjects [107].

AMD = age-related macular degeneration; EPA = eicosapentaenoic acid; DHA = docosahexaenoic acid; MPOD = macular pigment optical density.

**Table A5:** Relationship between oral supplementation with lutein (±zeaxanthin) and lutein plasma concentration, MPOD levels, and visual performance in AMD patients.

**Lutein serum levels**	Significant increase in lutein serum levels [109,110,111,112,113]
**MPOD**	Significant increase [109,110,114,115,116,117,118,119]
	No significant increase [111,112,120,121]
**BCVA**	Significant improvement/stabilization [109,114,117]
	No significant improvement/stabilization [110,115,116,119,121]
**Contrast sensitivity**	Significant improvement [110,114,116,118,121]
	No significant improvement [116,123]
**Glare recovery time**	Significant reduction [114]
	No significant reduction [110]
**Microperimetric retinal sensitivity**	Significant increase [115]
	No significant increase [119]
**Multifocal ERG retinal sensitivity**	Significant increase [116,122]

MPOD = macular pigment optical density; BCVA = best-corrected visual acuity; ERG = electroretinogram.

**Table A6:** Relationship between dietary supplementation with specific nutrients and AMD risk.

Supplements	Effects
AREDS formula (500 mg of vitamins C, 400 IU of vitamin E, 15 mg of beta-carotene, 80 mg of zinc and 2 mg of copper/daily) [76]	Did not prevent AMD onset [48] 25% lower risk of AMD progression to late stages [both dry and wet forms] [76]
AREDS 2 formula (500 mg of vitamins C, 400 IU of vitamin E, 25 mg of zinc, 2 mg of copper, 10 mg of lutein, 2 mg of zeaxanthin/daily) [14]	Did not prevent AMD onset [48] 31% lower risk of AMD progression to late stages [both dry and wet forms] [14] Lower risk of GA progression of GA towards the fovea [124]
Lutein	Did not prevent AMD onset [48] Possible lower risk of AMD progression [109,115] with very low certainty of evidence [51]
Lutein ± zeaxanthin	Did not prevent AMD onset [48] Possible lower risk of AMD progression [115,116] with very low certainty of evidence [51]
Lutein + other antioxidants	Did not prevent AMD onset [48] Possible lower risk of AMD progression [114], with low certainty of evidence [51]
EPA, DHA, Vitamin E	Little or no effect on AMD progression [51]
Vitamin C, vitamin E, β-carotene	Did not prevent AMD onset [48]
Centrum Silver formula (15 mg of zinc, 45 IU of vitamin E, 60 mg of vitamin C, 5000 IU of β-carotene, 10 mg of vitamin A, 2.5 mg of folic acid, 50 mg of vitamin B6, 1 mg of vitamin B12)	Higher risk of AMD onset [48]
Zinc, folate, curcumin, saffron, goji berry	Under investigation

AMD = age-related macular degeneration; AREDS = Age-Related Eye Disease Study; IU = International Units; EPA = eicosapentaenoic acid; DHA = docosahexaenoic acid; GA = geographic atrophy.

## References

[1] Guymer R.H., Campbell T.G. (2023). Age-related macular degeneration. Lancet.

[2] Fleckenstein M., Schmitz-Valckenberg S., Chakravarthy U. (2024). Age-Related Macular Degeneration: A Review. JAMA.

[3] Keenan T.D.L., Cukras C.A., Chew E.Y. (2021). Age-Related Macular Degeneration: Epidemiology and Clinical Aspects. Adv. Exp. Med. Biol..

[4] Chaudhuri M., Hassan Y., Bakka Vemana P.P.S., Bellary Pattanashetty M.S., Abdin Z.U., Siddiqui H.F. (2023). Age-Related Macular Degeneration: An Exponentially Emerging Imminent Threat of Visual Impairment and Irreversible Blindness. Cureus.

[5] Deng Y., Qiao L., Du M., Qu C., Wan L., Li J., Huang L. (2021). Age-related macular degeneration: Epidemiology, genetics, pathophysiology, diagnosis, and targeted therapy. Genes Dis..

[6] Garcia-Garcia J., Usategui-Martin R., Sanabria M.R., Fernandez-Perez E., Telleria J.J., Coco-Martin R.M. (2022). Pathophysiology of Age-Related Macular Degeneration: Implications for Treatment. Ophthalmic Res..

[7] Terao J. (2023). Revisiting carotenoids as dietary antioxidants for human health and disease prevention. Food Funct..

[8] Arunkumar R., Gorusupudi A., Bernstein P.S. (2020). The macular carotenoids: A biochemical overview. Biochim. Biophys. Acta Mol. Cell Biol. Lipids.

[9] Demmig-Adams B., López-Pozo M., Stewart J.J., Adams W.W. (2020). Zeaxanthin and Lutein: Photoprotectors, Anti-Inflammatories, and Brain Food. Molecules.

[10] Jiang H., Wang L., Li J., Fan Y., Li Z., Ma M., Liu S., Li B., Shi J., Li C. (2023). Dietary vitamins, carotenoids and their sources in relation to age-related macular degeneration risk in China: A population-based case-control study. Br. J. Nutr..

[11] Joachim N., Kifley A., Colijn J.M., Lee K.E., Buitendijk G.H.S., Klein B.E.K., Myers C., Meuer S.M., Tan A.G., Flood V. (2018). Joint Contribution of Genetic Susceptibility and Modifiable Factors to the Progression of Age-Related Macular Degeneration over 10 Years: The Three Continent AMD Consortium Report. Ophthalmol. Retin..

[12] Li X., Holt R.R., Keen C.L., Morse L.S., Zivkovic A.M., Yiu G., Hackman R.M. (2023). Potential roles of dietary zeaxanthin and lutein in macular health and function. Nutr. Rev..

[13] Ye J., Cheng J., Xiong R., Chen H., Huang S., Li H., Pang J., Zhang X., Zhu H. (2024). Effects and Mechanisms of Lutein on Aging and Age-Related Diseases. Antioxidants.

[14] Age-Related Eye Disease Study 2 Research Group (2013). Lutein + zeaxanthin and omega-3 fatty acids for age-related macular degeneration: The Age-Related Eye Disease Study 2 (AREDS2) randomized clinical trial. JAMA.

[15] Feng L., Nie K., Jiang H., Fan W. (2019). Effects of lutein supplementation in age-related macular degeneration. PLoS ONE.

[16] Liu Y., Ni M., Wu R., Yang Z., Zhu X., Chen J. (2022). The level and efficacy of lutein in patients with age-related macular degeneration: A comprehensive systematic review and meta-analysis. Ann. Transl. Med..

[17] Böhm V., Lietz G., Olmedilla-Alonso B., Phelan D., Reboul E., Bánati D., Borel P., Corte-Real J., de Lera A.R., Desmarchelier C. (2021). From carotenoid intake to carotenoid blood and tissue concentrations—Implications for dietary intake recommendations. Nutr. Rev..

[18] Hughes L., Maurice D.M. (1984). A fresh look at iontophoresis. Arch. Ophthalmol..

[19] Singh P., Maibach H.I. (1994). Iontophoresis in drug delivery: Basic principles and applications. Crit. Rev. Ther. Drug Carr. Syst..

[20] Karpiński T.M. (2018). Selected Medicines Used in Iontophoresis. Pharmaceutics.

[21] Myles M.E., Neumann D.M., Hill J.M. (2005). Recent progress in ocular drug delivery for posterior segment disease: Emphasis on transscleral iontophoresis. Adv. Drug Deliv. Rev..

[22] Eljarrat-Binstock E., Domb A.J. (2006). Iontophoresis: A non-invasive ocular drug delivery. J. Control Release.

[23] Gratieri T., Santer V., Kalia Y.N. (2017). Basic principles and current status of transcorneal and transscleral iontophoresis. Expert Opin. Drug Deliv..

[24] Perez V.L., Wirostko B., Korenfeld M., From S., Raizman M. (2020). Ophthalmic Drug Delivery Using Iontophoresis: Recent Clinical Applications. J. Ocul. Pharmacol. Ther..

[25] Wei D., Pu N., Li S.Y., Wang Y.G., Tao Y. (2023). Application of iontophoresis in ophthalmic practice: An innovative strategy to deliver drugs into the eye. Drug Deliv..

[26] Sousa-Martins D., Sousa S., Duarte J., Marta M., Lombardo M., Lombardo G. (2016). Lutein reaches the retina following iontophoresis application. Investig. Ophthalmol. Vis. Sci..

[27] Lombardo M., Villari V., Micali N., Roy P., Sousa S.H., Lombardo G. (2018). Assessment of trans-scleral iontophoresis delivery of lutein to the human retina. J. Biophotonics.

[28] Lombardo M., Serrao S., Lombardo G. (2022). Challenges in Age-Related Macular Degeneration: From Risk Factors to Novel Diagnostics and Prevention Strategies. Front. Med..

[29] Mastropasqua R., Quarta A., Passamonti M., Lorenzi C., Ruggeri M.L., Gironi M., Persavalli C., Belloni Baroni L., De Santis Ciacci C., Aloia R. (2026). Macular pigment optical density following lutein scleral iontophoresis in intermediate age-related macular degeneration: A six-month pilot study. Br. J. Ophthalmol..

[30] Rinaldi M., Cennamo G., Fioretto G., Passaro M.L., De Falco F., Bertocchi M., Marotta M., Chiosi F., Calabro F., Strianese D. (2026). Scleral iontophoresis for targeted lutein delivery in intermediate stage dry AMD: Is this a new frontier in age macular degeneration?. Photodiagnosis Photodyn. Ther..

[31] Rinaldi M., Cennamo G., Passaro M.L., Chiosi F., Falco F., D’Alessandro A., Strianese D., Costagliola C. (2026). Targeting Macular Pigment in Intermediate Age-Related Macular Degeneration: Oral Supplementation Versus Transscleral Iontophoresis in a Prospective Pilot Study. J. Clin. Med..

[32] Zhang S., Ren J., Chai R., Yuan S., Hao Y. (2024). Global burden of low vision and blindness due to age-related macular degeneration from 1990 to 2021 and projections for 2050. BMC Public Health.

[33] Babaker R., Alzimami L., Al Ameer A., Almutairi M., Alam Aldeen R., Alshatti H., Al-Johani N., Al Taisan A. (2025). Risk factors for age-related macular degeneration: Updated systematic review and meta-analysis. Medicine.

[34] Tian M., Zhang B. (2025). Identification of risk factors for the progression of age-related macular degeneration: A systematic review and meta-analysis of cohort studies. Front. Med..

[35] Cougnard-Gregoire A., Merle B.M.J., Aslam T., Seddon J.M., Aknin I., Klaver C.C.W., Garhöfer G., Layana A.G., Minnella A.M., Silva R. (2023). Blue Light Exposure: Ocular Hazards and Prevention-A Narrative Review. Ophthalmol. Ther..

[36] Mrowicka M., Mrowicki J., Kucharska E., Majsterek I. (2022). Lutein and Zeaxanthin and Their Roles in Age-Related Macular Degeneration-Neurodegenerative Disease. Nutrients.

[37] Harris J., Wu D. (2025). The Role of Inflammation in Age-Related Macular Degeneration. Int. Ophthalmol. Clin..

[38] Cvekl A., Vijg J. (2024). Aging of the eye: Lessons from cataracts and age-related macular degeneration. Ageing Res. Rev..

[39] Nguyen J., Brantley M.A., Schwartz S.G. (2024). Genetics and Age-Related Macular Degeneration: A Practical Review for Clinicians. Front. Biosci..

[40] Hansman D.S., Du J., Casson R.J., Peet D.J. (2025). Eye on the horizon: The metabolic landscape of the RPE in aging and disease. Prog. Retin. Eye Res..

[41] Śpiewak D., Drzyzga Ł., Dorecka M., Wyględowska-Promieńska D. (2024). Summary of the Therapeutic Options for Patients with Dry and Neovascular AMD. J. Clin. Med..

[42] Yin X., He T., Yang S., Cui H., Jiang W. (2022). Efficacy and Safety of Antivascular Endothelial Growth Factor (Anti-VEGF) in Treating Neovascular Age-Related Macular Degeneration (AMD): A Systematic Review and Meta-analysis. J. Immunol. Res..

[43] Cabral de Guimaraes T.A., Daich Varela M., Georgiou M., Michaelides M. (2022). Treatments for dry age-related macular degeneration: Therapeutic avenues, clinical trials and future directions. Br. J. Ophthalmol..

[44] Girgis S., Lee L.R. (2023). Treatment of dry age-related macular degeneration: A review. Clin. Exp. Ophthalmol..

[45] Marques-Couto P., Coelho-Costa I., Ferreira-da-Silva R., Andrade J.P., Carneiro Â. (2025). Mediterranean Diet on Development and Progression of Age-Related Macular Degeneration: Systematic Review and Meta-Analysis of Observational Studies. Nutrients.

[46] Hu P., He M., Cai J., Lin Z., Zheng S., Zhuang Z., Liu J., Huang L. (2025). Global burden of smoking-associated age-related macular degeneration: Spatiotemporal trends from 1990 to 2021 and projections to 2040. Tob. Induc. Dis..

[47] Mauschitz M.M., Schmitz M.T., Verzijden T., Schmid M., Thee E.F., Colijn J.M., Delcourt C., Cougnard-Grégoire A., Merle B.M.J., Korobelnik J.F. (2022). European Eye Epidemiology (E3) Consortium. Physical Activity, Incidence, and Progression of Age-Related Macular Degeneration: A Multicohort Study. Am. J. Ophthalmol..

[48] Evans J.R., Lawrenson J.G. (2017). Antioxidant vitamin and mineral supplements for preventing age-related macular degeneration. Cochrane Database Syst. Rev..

[49] Csader S., Korhonen S., Kaarniranta K., Schwab U. (2022). The Effect of Dietary Supplementations on Delaying the Progression of Age-Related Macular Degeneration: A Systematic Review and Meta-Analysis. Nutrients.

[50] Pameijer E.M., Heus P., Damen J.A.A., Spijker R., Hooft L., Ringens P.J., Imhof S.M., van Leeuwen R. (2022). What did we learn in 35 years of research on nutrition and supplements for age-related macular degeneration: A systematic review. Acta Ophthalmol..

[51] Evans J.R., Lawrenson J.G. (2023). Antioxidant vitamin and mineral supplements for slowing the progression of age-related macular degeneration. Cochrane Database Syst. Rev..

[52] Parmar U.P.S., Surico P.L., Mori T., Singh R.B., Cutrupi F., Premkishore P., Gallo Afflitto G., Di Zazzo A., Coassin M., Romano F. (2025). Antioxidants in Age-Related Macular Degeneration: Lights and Shadows. Antioxidants.

[53] Hahn P., Eichenbaum D., Dhoot D.S., Wykoff C.C., Klufas M.A., Intorcia M., Jones D., Sarda S.P., Bobbili P., Chang R. (2025). Efficacy of Intravitreal Pegcetacoplan vs Avacincaptad Pegol in Patients With Geographic Atrophy. J. Vitreoretin. Dis..

[54] Kiser P.D. (2022). Retinal pigment epithelium 65 kDa protein (RPE65): An update. Prog. Retin. Eye Res..

[55] Li L.H., Lee J.C., Leung H.H., Lam W.C., Fu Z., Lo A.C.Y. (2020). Lutein Supplementation for Eye Diseases. Nutrients.

[56] Bhat I., Mamatha B.S. (2021). Genetic factors involved in modulating lutein bioavailability. Nutr. Res..

[57] Figueiredo I., Farinha C., Barreto P., Coimbra R., Pereira P., Marques J.P., Pires I., Cachulo M.L., Silva R. (2024). Nutritional Genomics: Implications for Age-Related Macular Degeneration. Nutrients.

[58] Ceravolo S.A., Hammond B.R., Oliver W., Clementz B., Miller L.S., Renzi-Hammond L.M. (2019). Dietary Carotenoids Lutein and Zeaxanthin Change Brain Activation in Older Adult Participants: A Randomized, Double-Masked, Placebo-Controlled Trial. Mol. Nutr. Food Res..

[59] Lopresti A.L., Smith S.J., Drummond P.D. (2022). The Effects of Lutein and Zeaxanthin Supplementation on Cognitive Function in Adults With Self-Reported Mild Cognitive Complaints: A Randomized, Double-Blind, Placebo-Controlled Study. Front. Nutr..

[60] He X., Yin X., Chen X., Chen X. (2023). Aging and antioxidants: The impact of dietary carotenoid intakes on soluble klotho levels in aged adults. Front. Endocrinol..

[61] Masri A., Armanazi M., Inouye K., Geierhart D.L., Davey P.G., Vasudevan B. (2024). Macular Pigment Optical Density as a Measurable Modifiable Clinical Biomarker. Nutrients.

[62] Bone R.A., Landrum J.T. (2004). Heterochromatic flicker photometry. Arch. Biochem. Biophys..

[63] Davey P.G., Rosen R.B., Gierhart D.L. (2021). Macular Pigment Reflectometry: Developing Clinical Protocols, Comparison with Heterochromatic Flicker Photometry and Individual Carotenoid Levels. Nutrients.

[64] Udensi J., Loughman J., Loskutova E., Byrne H.J. (2022). Raman Spectroscopy of Carotenoid Compounds for Clinical Applications-A Review. Molecules.

[65] Ma L., Liu R., Du J.H., Liu T., Wu S.S., Liu X.H. (2016). Lutein, Zeaxanthin and Meso-zeaxanthin Supplementation Associated with Macular Pigment Optical Density. Nutrients.

[66] Bone R.A., Davey P.G., Roman B.O., Evans D.W. (2020). Efficacy of Commercially Available Nutritional Supplements: Analysis of Serum Uptake, Macular Pigment Optical Density and Visual Functional Response. Nutrients.

[67] Wilson L.M., Tharmarajah S., Jia Y., Semba R.D., Schaumberg D.A., Robinson K.A. (2021). The Effect of Lutein/Zeaxanthin Intake on Human Macular Pigment Optical Density: A Systematic Review and Meta-Analysis. Adv. Nutr..

[68] Loughman J., Akkali M.C., Beatty S., Scanlon G., Davison P.A., O’Dwyer V., Cantwell T., Major P., Stack J., Nolan J.M. (2010). The relationship between macular pigment and visual performance. Vis. Res..

[69] Machida N., Kosehira M., Kitaichi N. (2020). Clinical Effects of Dietary Supplementation of Lutein with High Bio-Accessibility on Macular Pigment Optical Density and Contrast Sensitivity: A Randomized Double-Blind Placebo-Controlled Parallel-Group Comparison Trial. Nutrients.

[70] Johnson E.J., Avendano E.E., Mohn E.S., Raman G. (2021). The association between macular pigment optical density and visual function outcomes: A systematic review and meta-analysis. Eye.

[71] Richer S., Novil S., Gullett T., Dervishi A., Nassiri S., Duong C., Davis R., Davey P.G. (2021). Night Vision and Carotenoids (NVC): A Randomized Placebo Controlled Clinical Trial on Effects of Carotenoid Supplementation on Night Vision in Older Adults. Nutrients.

[72] Wysocky C.J., Harth J.B., Renzi-Hammond L.M., Hammond B.R. (2026). The relationship between the macular carotenoids and simulated visual range. Exp. Eye Res..

[73] Roark M.W., Stringham J.M. (2019). Visual Performance in the “Real World”: Contrast Sensitivity, Visual Acuity, and Effects of Macular Carotenoids. Mol. Nutr. Food Res..

[74] Hu W., Shankar P., Yao Y., Su X., Kim J.E. (2023). Effect of xanthophyll-rich food and supplement intake on visual outcomes in healthy adults and those with eye disease: A systematic review, meta-analysis, and meta-regression of randomized controlled trials. Nutr. Rev..

[75] Obana A., Gohto Y., Tanito M., Okazaki S., Gellermann W., Bernstein P.S., Ohira A. (2014). Effect of age and other factors on macular pigment optical density measured with resonance Raman spectroscopy. Graefes Arch. Clin. Exp. Ophthalmol..

[76] Age-Related Eye Disease Study Research Group (2001). A randomized, placebo-controlled, clinical trial of high-dose supplementation with vitamins C and E, beta carotene, and zinc for age-related macular degeneration and vision loss: AREDS report no. 8. Arch. Ophthalmol..

[77] Lem D.W., Davey P.G., Gierhart D.L., Rosen R.B. (2021). A Systematic Review of Carotenoids in the Management of Age-Related Macular Degeneration. Antioxidants.

[78] Dinu M., Pagliai G., Casini A., Sofi F. (2019). Food groups and risk of age-related macular degeneration: A systematic review with meta-analysis. Eur. J. Nutr..

[79] Agrón E., Mares J., Clemons T.E., Swaroop A., Chew E.Y., Keenan T.D.L. (2021). AREDS and AREDS2 Research Groups. Dietary Nutrient Intake and Progression to Late Age-Related Macular Degeneration in the Age-Related Eye Disease Studies 1 and 2. Ophthalmology.

[80] Keenan T.D., Agrón E., Mares J., Clemons T.E., van Asten F., Swaroop A., Chew E.Y. (2020). Age-Related Eye Disease Studies (AREDS) 1 and 2 Research Groups. Adherence to the Mediterranean Diet and Progression to Late Age-Related Macular Degeneration in the Age-Related Eye Disease Studies 1 and 2. Ophthalmology.

[81] Zhong Y., Wang K., Jiang L., Wang J., Zhang X., Xu J., Yao K. (2021). Dietary fatty acid intake, plasma fatty acid levels, and the risk of age-related macular degeneration (AMD): A dose-response meta-analysis of prospective cohort studies. Eur. J. Nutr..

[82] Moeller S.M., Parekh N., Tinker L., Ritenbaugh C., Blodi B., Wallace R.B., Mares J.A. (2006). CAREDS Research Study Group. Associations between intermediate age-related macular degeneration and lutein and zeaxanthin in the Carotenoids in Age-related Eye Disease Study (CAREDS): Ancillary study of the Women’s Health Initiative. Arch. Ophthalmol..

[83] Tan J.S., Wang J.J., Flood V., Rochtchina E., Smith W., Mitchell P. (2008). Dietary antioxidants and the long-term incidence of age-related macular degeneration: The Blue Mountains Eye Study. Ophthalmology.

[84] Ho L., van Leeuwen R., Witteman J.C., van Duijn C.M., Uitterlinden A.G., Hofman A., de Jong P.T., Vingerling J.R., Klaver C.C. (2011). Reducing the genetic risk of age-related macular degeneration with dietary antioxidants, zinc, and ω-3 fatty acids: The Rotterdam study. Arch. Ophthalmol..

[85] Stevens R., Bartlett H., Cooke R. (2015). Dietary analysis and nutritional behaviour in people with and without age-related macular disease. Clin. Nutr. ESPEN.

[86] Wu J., Cho E., Willett W.C., Sastry S.M., Schaumberg D.A. (2015). Intakes of Lutein, Zeaxanthin, and Other Carotenoids and Age-Related Macular Degeneration During 2 Decades of Prospective Follow-up. JAMA Ophthalmol..

[87] Arslan S., Kadayifçilar S., Samur G. (2019). The Potential Role of Dietary Antioxidant Capacity in Preventing Age-Related Macular Degeneration. J. Am. Coll. Nutr..

[88] Mares-Perlman J.A., Fisher A.I., Klein R., Palta M., Block G., Millen A.E., Wright J.D. (2001). Lutein and zeaxanthin in the diet and serum and their relation to age-related maculopathy in the third national health and nutrition examination survey. Am. J. Epidemiol..

[89] Cho E., Hankinson S.E., Rosner B., Willett W.C., Colditz G.A. (2008). Prospective study of lutein/zeaxanthin intake and risk of age-related macular degeneration. Am. J. Clin. Nutr..

[90] Lin H., Mares J.A., LaMonte M.J., Brady W.E., Sahli M.W., Klein R., Klein B.E.K., Nie J., Millen A.E. (2017). Association between Dietary Xanthophyll (Lutein and Zeaxanthin) Intake and Early Age-Related Macular Degeneration: The Atherosclerosis Risk in Communities Study. Ophthalmic Epidemiol..

[91] Delcourt C., Carrière I., Delage M., Barberger-Gateau P., Schalch W. (2006). POLA Study Group. Plasma lutein and zeaxanthin and other carotenoids as modifiable risk factors for age-related maculopathy and cataract: The POLA Study. Investig. Ophthalmol. Vis. Sci..

[92] Ng A.L., Leung H.H., Kawasaki R., Ho W.L., Chow L.L., Chow S.S., Lee J.C., Wong I.Y. (2019). Dietary Habits, Fatty Acids and Carotenoid Levels Are Associated with Neovascular Age-Related Macular Degeneration in Chinese. Nutrients.

[93] Jiang H., Fan Y., Li J., Wang J., Kong L., Wang L., Li Z., Ma M., Shi X., Liu S. (2022). The Associations of Plasma Carotenoids and Vitamins With Risk of Age-Related Macular Degeneration: Results From a Matched Case-Control Study in China and Meta-Analysis. Front. Nutr..

[94] Li Z., Li Y., Hou Y., Fan Y., Jiang H., Li B., Zhu H., Liu Y., Zhang L., Zhang J. (2023). Association of Plasma Vitamins and Carotenoids, DNA Methylation of LCAT, and Risk of Age-Related Macular Degeneration. Nutrients.

[95] Michikawa T., Ishida S., Nishiwaki Y., Kikuchi Y., Tsuboi T., Hosoda K., Ishigami A., Iwasawa S., Nakano M., Takebayashi T. (2009). Serum antioxidants and age-related macular degeneration among older Japanese. Asia Pac. J. Clin. Nutr..

[96] Merle B.M.J., Cougnard-Grégoire A., Korobelnik J.F., Schalch W., Etheve S., Rougier M.B., Féart C., Samieri C., Delyfer M.N., Delcourt C. (2021). Plasma Lutein, a Nutritional Biomarker for Development of Advanced Age-Related Macular Degeneration: The Alienor Study. Nutrients.

[97] Dasch B., Fuhs A., Schmidt J., Behrens T., Meister A., Wellmann J., Fobker M., Pauleikhoff D., Hense H.W. (2005). Serum levels of macular carotenoids in relation to age-related maculopathy: The Muenster Aging and Retina Study (MARS). Graefe’s Arch. Clin. Exp. Ophthalmol..

[98] Obana A., Hiramitsu T., Gohto Y., Ohira A., Mizuno S., Hirano T., Bernstein P.S., Fujii H., Iseki K., Tanito M. (2008). Macular carotenoid levels of normal subjects and age-related maculopathy patients in a Japanese population. Ophthalmology.

[99] Kaya S., Weigert G., Pemp B., Sacu S., Werkmeister R.M., Dragostinoff N., Garhöfer G., Schmidt-Erfurth U., Schmetterer L. (2012). Comparison of macular pigment in patients with age-related macular degeneration and healthy control subjects—A study using spectral fundus reflectance. Acta Ophthalmol..

[100] Nagai N., Minami S., Suzuki M., Shinoda H., Kurihara T., Sonobe H., Watanabe K., Uchida A., Ban N., Tsubota K. (2020). Macular Pigment Optical Density and Photoreceptor Outer Segment Length as Predisease Biomarkers for Age-Related Macular Degeneration. J. Clin. Med..

[101] Çalış Karanfil F., Özmert E. (2025). Evaluation of the macula pigment optical density by a psychophysical test in dry age-related macular degeneration. Eur. J. Ophthalmol..

[102] Sayin O., Altinkaynak H. (2023). Macular Pigment Optical Density in First Degree Relatives of Age-Related Macular Degeneration Patients. Curr. Eye Res..

[103] Arunkumar R., Calvo C.M., Conrady C.D., Bernstein P.S. (2018). What do we know about the macular pigment in AMD: The past, the present, and the future. Eye.

[104] Tsika C., Tsilimbaris M.K., Makridaki M., Kontadakis G., Plainis S., Moschandreas J. (2011). Assessment of macular pigment optical density (MPOD) in patients with unilateral wet age-related macular degeneration (AMD). Acta Ophthalmol..

[105] Ren X.T., Gu H., Han X., Zhang J.Y., Li X., Yang X.F., Xu J., Snellingen T., Liu X.P., Wang N.L. (2015). Measurement of macular pigment optical density among healthy Chinese people and patients with early-stage age-related macular degeneration. Int. J. Ophthalmol..

[106] Ozyurt A., Kocak N., Akan P., Calan O.G., Ozturk T., Kaya M., Karahan E., Kaynak S. (2017). Comparison of macular pigment optical density in patients with dry and wet age-related macular degeneration. Indian J. Ophthalmol..

[107] Goerdt L., Berlin A., Gao L., Swain T.A., Kim S.S., McGwin G., Clark M.E., Kar D., Owsley C., Sloan K.R. (2025). Topographic Analysis of Two-Wavelength Autofluorescence Supports Higher Macular Xanthophyll Pigment in AMD Than Aging: ALSTAR2 Baseline. Investig. Ophthalmol. Vis. Sci..

[108] Alharbi A.M., Kilani M.A., Berendschot T.T. (2021). Overflow phenomenon in serum lutein after supplementation: A systematic review supported with SNPs analyses. Int. J. Ophthalmol..

[109] Murray I.J., Makridaki M., van der Veen R.L., Carden D., Parry N.R., Berendschot T.T. (2013). Lutein supplementation over a one-year period in early AMD might have a mild beneficial effect on visual acuity: The CLEAR study. Investig. Ophthalmol. Vis. Sci..

[110] Huang Y.M., Dou H.L., Huang F.F., Xu X.R., Zou Z.Y., Lin X.M. (2015). Effect of supplemental lutein and zeaxanthin on serum, macular pigmentation, and visual performance in patients with early age-related macular degeneration. Biomed. Res. Int..

[111] Korobelnik J.F., Rougier M.B., Delyfer M.N., Bron A., Merle B.M.J., Savel H., Chêne G., Delcourt C., Creuzot-Garcher C. (2017). Effect of Dietary Supplementation With Lutein, Zeaxanthin, and ω-3 on Macular Pigment: A Randomized Clinical Trial. JAMA Ophthalmol..

[112] Sawa M., Shunto T., Nishiyama I., Yokoyama A., Shigeta R., Miura S., Kawasaki R. (2020). Effects of Lutein Supplementation in Japanese Patients with Unilateral Age-Related Macular Degeneration: The Sakai Lutein Study. Sci. Rep..

[113] García-Layana A., Recalde S., Hernandez M., Abraldes M.J., Nascimento J., Hernández-Galilea E., Olmedilla-Alonso B., Escobar-Barranco J.J., Zapata M.A., Silva R. (2021). A Randomized Study of Nutritional Supplementation in Patients with Unilateral Wet Age-Related Macular Degeneration. Nutrients.

[114] Richer S., Stiles W., Statkute L., Pulido J., Frankowski J., Rudy D., Pei K., Tsipursky M., Nyland J. (2004). Double-masked, placebo-controlled, randomized trial of lutein and antioxidant supplementation in the intervention of atrophic age-related macular degeneration: The Veterans LAST study (Lutein Antioxidant Supplementation Trial). Optometry.

[115] Weigert G., Kaya S., Pemp B., Sacu S., Lasta M., Werkmeister R.M., Dragostinoff N., Simader C., Garhöfer G., Schmidt-Erfurth U. (2011). Effects of lutein supplementation on macular pigment optical density and visual acuity in patients with age-related macular degeneration. Investig. Ophthalmol. Vis. Sci..

[116] Ma L., Yan S.F., Huang Y.M., Lu X.R., Qian F., Pang H.L., Xu X.R., Zou Z.Y., Dong P.C., Xiao X. (2012). Effect of lutein and zeaxanthin on macular pigment and visual function in patients with early age-related macular degeneration. Ophthalmology.

[117] Dawczynski J., Jentsch S., Schweitzer D., Hammer M., Lang G.E., Strobel J. (2013). Long term effects of lutein, zeaxanthin and omega-3-LCPUFAs supplementation on optical density of macular pigment in AMD patients: The LUTEGA study. Graefes Arch. Clin. Exp. Ophthalmol..

[118] Wolf-Schnurrbusch U.E., Zinkernagel M.S., Munk M.R., Ebneter A., Wolf S. (2015). Oral Lutein Supplementation Enhances Macular Pigment Density and Contrast Sensitivity but Not in Combination With Polyunsaturated Fatty Acids. Investig. Ophthalmol. Vis. Sci..

[119] Corvi F., Souied E.H., Falfoul Y., Georges A., Jung C., Querques L., Querques G. (2017). Pilot evaluation of short-term changes in macular pigment and retinal sensitivity in different phenotypes of early age-related macular degeneration after carotenoid supplementation. Br. J. Ophthalmol..

[120] Azar G., Quaranta-El Maftouhi M., Masella J.J., Mauget-Faÿsse M. (2017). Macular pigment density variation after supplementation of lutein and zeaxanthin using the Visucam^®^ 200 pigment module: Impact of age-related macular degeneration and lens status. J. Fr. Ophtalmol..

[121] Davey P.G., Henderson T., Lem D.W., Weis R., Amonoo-Monney S., Evans D.W. (2020). Visual Function and Macular Carotenoid Changes in Eyes with Retinal Drusen-An Open Label Randomized Controlled Trial to Compare a Micronized Lipid-Based Carotenoid Liquid Supplementation and AREDS-2 Formula. Nutrients.

[122] Huang Y.M., Dou H.L., Huang F.F., Xu X.R., Zou Z.Y., Lu X.R., Lin X.M. (2015). Changes following supplementation with lutein and zeaxanthin in retinal function in eyes with early age-related macular degeneration: A randomised, double-blind, placebo-controlled trial. Br. J. Ophthalmol..

[123] Bartlett H.E., Eperjesi F. (2007). Effect of lutein and antioxidant dietary supplementation on contrast sensitivity in age-related macular disease: A randomized controlled trial. Eur. J. Clin. Nutr..

[124] Keenan T.D.L., Agrón E., Keane P.A., Domalpally A., Chew E.Y. (2025). Age-Related Eye Disease Study Research Group; Age-Related Eye Disease Study 2 Research Group. Oral Antioxidant and Lutein/Zeaxanthin Supplements Slow Geographic Atrophy Progression to the Fovea in Age-Related Macular Degeneration. Ophthalmology.

[125] Choi R.Y., Chortkoff S.C., Gorusupudi A., Bernstein P.S. (2016). Crystalline Maculopathy Associated With High-Dose Lutein Supplementation. JAMA Ophthalmol..

[126] Ranard K.M., Jeon S., Mohn E.S., Griffiths J.C., Johnson E.J., Erdman J.W. (2017). Dietary guidance for lutein: Consideration for intake recommendations is scientifically supported. Eur. J. Nutr..

[127] Shao A., Hathcock J.N. (2006). Risk assessment for the carotenoids lutein and lycopene. Regul. Toxicol. Pharmacol..

[128] Black H.S., Boehm F., Edge R., Truscott T.G. (2020). The Benefits and Risks of Certain Dietary Carotenoids that Exhibit both Anti- and Pro-Oxidative Mechanisms-A Comprehensive Review. Antioxidants.

[129] Dosmar E., Walsh J., Doyel M., Bussett K., Oladipupo A., Amer S., Goebel K. (2022). Targeting Ocular Drug Delivery: An Examination of Local Anatomy and Current Approaches. Bioengineering.

[130] Ahmed S., Amin M.M., Sayed S. (2023). Ocular Drug Delivery: A Comprehensive Review. AAPS PharmSciTech.

[131] Kang-Mieler J.J., Rudeen K.M., Liu W., Mieler W.F. (2020). Advances in ocular drug delivery systems. Eye.

[132] Giri B.R., Jakka D., Sandoval M.A., Kulkarni V.R., Bao Q. (2024). Advancements in Ocular Therapy: A Review of Emerging Drug Delivery Approaches and Pharmaceutical Technologies. Pharmaceutics.

[133] Byrne J.D., Yeh J.J., DeSimone J.M. (2018). Use of iontophoresis for the treatment of cancer. J. Control Release.

[134] Ragit R., Fulzele P., Rathi N.V., Thosar N.R., Khubchandani M., Malviya N.S., Das S. (2022). Iontophoresis as an Effective Drug Delivery System in Dentistry: A Review. Cureus.

[135] Piscazzi F., D’Oria F., Ramirez M.A., Ardigò M. (2026). Iontophoresis-Based Topical Drug Delivery for Dermatologic Conditions: A Systematic Review. Pharmaceuticals.

[136] Bakshi P., Vora D., Hemmady K., Banga A.K. (2020). Iontophoretic skin delivery systems: Success and failures. Int. J. Pharm..

[137] Lam T.T., Fu J., Tso M.O. (1991). A histopathologic study of retinal lesions inflicted by transscleral iontophoresis. Graefes Arch. Clin. Exp. Ophthalmol..

[138] Behar-Cohen F.F., El Aouni A., Gautier S., David G., Davis J., Chapon P., Parel J.M. (2002). Transscleral Coulomb-controlled iontophoresis of methylprednisolone into the rabbit eye: Influence of duration of treatment, current intensity and drug concentration on ocular tissue and fluid levels. Exp. Eye Res..

[139] Patane M.A., Schubert W., Sanford T., Gee R., Burgos M., Isom W.P., Ruiz-Perez B. (2013). Evaluation of ocular and general safety following repeated dosing of dexamethasone phosphate delivered by transscleral iontophoresis in rabbits. J. Ocul. Pharmacol. Ther..

[140] Lee S., Lee C., Kim E., Ko S.A., Kim S.N., Choy Y.B., Im C.H. (2022). In-vivo estimation of tissue electrical conductivities of a rabbit eye for precise simulation of electric field distributions during ocular iontophoresis. Int. J. Numer. Method. Biomed. Eng..

[141] Hikima T., Ezaki H. (2023). Predicting the Safety of Ocular Iontophoresis Using Reconstructed Human Corneal Epithelial Cells. Biol. Pharm. Bull..

[142] Zibura A.E., Cullen M.A., Rutledge H., Lassalle L., Salmon J.H., Gilger B.C., Westermeyer H.D. (2020). Optimizing corneal riboflavin administration in ex vivo horse, dog, rabbit, and pig samples for use in corneal collagen cross-linking. Vet. Ophthalmol..

[143] Rong S., Wang C., Han B., Feng P., Lan W., Gao Z., Li X., Chen W. (2017). Iontophoresis-assisted accelerated riboflavin/ultraviolet A scleral cross-linking: A potential treatment for pathologic myopia. Exp. Eye Res..

[144] Barza M., Peckman C., Baum J. (1986). Transscleral iontophoresis of cefazolin, ticarcillin, and gentamicin in the rabbit. Ophthalmology.

[145] Choi T.B., Lee D.A. (1988). Transscleral and transcorneal iontophoresis of vancomycin in rabbit eyes. J. Ocul. Pharmacol..

[146] Vollmer D.L., Szlek M.A., Kolb K., Lloyd L.B., Parkinson T.M. (2002). In vivo transscleral iontophoresis of amikacin to rabbit eyes. J. Ocul. Pharmacol. Ther..

[147] Frucht-Pery J., Raiskup F., Mechoulam H., Shapiro M., Eljarrat-Binstock E., Domb A. (2006). Iontophoretic treatment of experimental pseudomonas keratitis in rabbit eyes using gentamicin-loaded hydrogels. Cornea.

[148] Vaka S.R., Sammeta S.M., Day L.B., Murthy S.N. (2008). Transcorneal iontophoresis for delivery of ciprofloxacin hydrochloride. Curr. Eye Res..

[149] Lam T.T., Fu J., Chu R., Stojack K., Siew E., Tso M.O. (1994). Intravitreal delivery of ganciclovir in rabbits by transscleral iontophoresis. J. Ocul. Pharmacol..

[150] Yoshizumi M.O., Roca J.A., Lee D.A., Lee G., Gomez I. (1996). Ocular iontophoretic supplementation of intravenous foscarnet therapy. Am. J. Ophthalmol..

[151] Chen Y., Kalia Y.N. (2018). Short-duration ocular iontophoresis of ionizable aciclovir prodrugs: A new approach to treat herpes simplex infections in the anterior and posterior segments of the eye. Int. J. Pharm..

[152] Grossman R., Lee D.A. (1989). Transscleral and transcorneal iontophoresis of ketoconazole in the rabbit eye. Ophthalmology.

[153] Kalkanci A., Yesilirmak N., Ozdemir H.B., Unal E.A., Erdoğan M., Seker T., Tum A.E., Karakus A.K., Hizel K., Bilgihan K. (2018). Impact of Iontophoresis and PACK-CXL Corneal Concentrations of Antifungals in an In Vivo Model. Cornea.

[154] González Iglesias L.G., Kalia Y.N. (2025). Ionto phoretic delivery of caspofungin acetate to the cornea and sclera and its intracorneal biodistribution. Int. J. Pharm. X.

[155] Eljarrat-Binstock E., Raiskup F., Frucht-Pery J., Domb A.J. (2005). Transcorneal and transscleral iontophoresis of dexamethasone phosphate using drug loaded hydrogel. J. Control Release.

[156] Santer V., Chen Y., Kalia Y.N. (2018). Controlled non-invasive iontophoretic delivery of triamcinolone acetonide amino acid ester prodrugs into the posterior segment of the eye. Eur. J. Pharm. Biopharm..

[157] Kralinger M.T., Voigt M., Kieselbach G., Hamasaki D., Hayden B., Parel J.M. (2003). Ocular delivery of acetylsalicylic acid by repetitive coulomb-controlled iontophoresis. Ophthalmic Res..

[158] Eljarrat-Binstock E., Domb A.J., Orucov F., Frucht-Pery J., Pe’er J. (2007). Methotrexate delivery to the eye using transscleral hydrogel iontophoresis. Curr. Eye Res..

[159] Souied E.H., Reid S.N., Piri N.I., Lerner L.E., Nusinowitz S., Farber D.B. (2008). Non-invasive gene transfer by iontophoresis for therapy of an inherited retinal degeneration. Exp. Eye Res..

[160] Hayden B., Jockovich M.E., Murray T.G., Kralinger M.T., Voigt M., Hernandez E., Feuer W., Parel J.M. (2006). Iontophoretic delivery of carboplatin in a murine model of retinoblastoma. Investig. Ophthalmol. Vis. Sci..

[161] Molokhia S., Papangkorn K., Butler C., Higuchi J.W., Brar B., Ambati B., Li S.K., Higuchi W.I. (2020). Transscleral Iontophoresis for Noninvasive Ocular Drug Delivery of Macromolecules. J. Ocul. Pharmacol. Ther..

[162] González Iglesias L.G., Messaoudi S., Kalia Y.N. (2022). Non-Invasive Iontophoretic Delivery of Cytochrome c to the Posterior Segment and Determination of Its Ocular Biodistribution. Pharmaceutics.

[163] Parkinson T.M., Ferguson E., Febbraro S., Bakhtyari A., King M., Mundasad M. (2003). Tolerance of ocular iontophoresis in healthy volunteers. J. Ocul. Pharmacol. Ther..

[164] Horwath-Winter J., Schmut O., Haller-Schober E.M., Gruber A., Rieger G. (2005). Iodide iontophoresis as a treatment for dry eye syndrome. Br. J. Ophthalmol..

[165] Patane M.A., Cohen A., From S., Torkildsen G., Welch D., Ousler G.W. (2011). Ocular iontophoresis of EGP-437 (dexamethasone phosphate) in dry eye patients: Results of a randomized clinical trial. Clin. Ophthalmol..

[166] Lombardo M., Giannini D., Lombardo G., Serrao S. (2017). Randomized Controlled Trial Comparing Transepithelial Corneal Cross-linking Using Iontophoresis with the Dresden Protocol in Progressive Keratoconus. Ophthalmology.

[167] Alqudah N., Alqudah A., Alshamarti S., Shannak Z. (2025). Evaluating the efficacy and safety of iontophoresis-assisted corneal cross-linking compared to standard CXL in the treatment of keratoconus. Indian J. Ophthalmol..

[168] Halhal M., Renard G., Bejjani R.A., Behar-Cohen F. (2003). Rejet de greffe et iontophorèse de corticoïdes: à propos de 3 cas [Corneal graft rejection and corticoid iontophoresis: 3 case reports]. J. Fr. Ophtalmol..

[169] O’Neil E.C., Huang J., Suhler E.B., Dunn J.P., Perez V.L., Gritz D.C., McWilliams K., Peskin E., Ying G.S., Bunya V.Y. (2018). Iontophoretic delivery of dexamethasone phosphate for non-infectious, non-necrotising anterior scleritis, dose-finding clinical trial. Br. J. Ophthalmol..

[170] Cohen A.E., Assang C., Patane M.A., From S., Korenfeld M., Avion Study Investigators (2012). Evaluation of dexamethasone phosphate delivered by ocular iontophoresis for treating noninfectious anterior uveitis. Ophthalmology.

[171] Sheppard J., Garg S., Lievens C., Brandano L., Wirostko B., Korenfeld M., Raizman M., Foster C.S. (2020). Iontophoretic Dexamethasone Phosphate Compared to Topical Prednisolone Acetate 1% for Noninfectious Anterior Segment Uveitis. Am. J. Ophthalmol..

[172] Bilmin K., Synoradzki K.J., Czarnecka A.M., Spałek M.J., Kujawska T., Solnik M., Merks P., Toro M.D., Rejdak R., Fiedorowicz M. (2021). New Perspectives for Eye-Sparing Treatment Strategies in Primary Uveal Melanoma. Cancers.

[173] Monés J., Pagani F., Santmaría J.F., Garcia M., Romero C., Garcia D., Serrano A., Carrasco A. (2025). Spontaneous Soft Drusen Regression without Atrophy and the Drusen Ooze. Ophthalmol. Retin..

